# Explicit discrimination and ingroup favoritism, but no implicit biases in hypothetical triage decisions during COVID-19

**DOI:** 10.1038/s41598-023-50385-w

**Published:** 2024-01-12

**Authors:** Nico Gradwohl, Hansjörg Neth, Helge Giese, Wolfgang Gaissmaier

**Affiliations:** 1 Department of Psychology, University of Konstanz, Konstanz, Germany; 2 Cluster of Excellence “Centre for the Advanced Study of Collective Behaviour”, University of Konstanz, Konstanz, Germany; 3 Department for the Ecology of Animal Societies, Max Planck Institute of Animal Behavior, Konstanz, Germany; 4 Charité – Universitätsmedizin Berlin, Berlin, Germany

**Keywords:** Psychology, Human behaviour

## Abstract

Disturbingly realistic triage scenarios during the COVID-19 pandemic provide an opportunity for studying discrimination in moral reasoning. Biases and favoritism do not need to be explicit and overt, but can remain implicit and covert. In addition to assessing laypeople’s propensity for engaging in overt discrimination, the present study examines whether they reveal implicit biases through seemingly fair random allocations. We present a cross-sectional online study comprising 6 timepoints and a total of 2296 participants. Each individual evaluated 19 hypothetical scenarios that provide an allocation dilemma between two patients who are in need of ventilation and differ only in one focal feature. Participants could either allocate the last ventilator to a patient, or opt for random allocation to express impartiality. Overall, participants exhibited clear biases for the patient who was expected to be favored based on health factors, previous ethical or caretaking behaviors, and in-group favoritism. If one patient had been pre-allocated care, a higher probability of keeping the ventilator for the favored patient indicates persistent favoritism. Surprisingly, the absence of an asymmetry in random allocations indicates the absence of covert discrimination. Our results demonstrate that laypeople’s hypothetical triage decisions discriminate overtly and show explicit biases.

## Introduction

A shortage of intensive care capacities during the COVID-19 pandemic forced doctors to make disturbing decisions in which they had to prioritize which patient to save^1,2^. Laypeople’s decisions in such triage scenarios^3^ provide an opportunity for studying moral reasoning in less artificial contexts than traditional sacrificial dilemmas^4–7^, such as the trolley dilemma^8,9^. Recent ethical guidelines for medical situations of extreme scarcity are mostly based on utilitarian considerations. To save the largest number of lives, they advocate saving those patients with the best prognosis^2^. In the absence of relevant distinctions, ethical guidelines recommend a random allocation of resources^1,10^ (but see^11^). Laypeople largely endorse such guidelines, but have been found to deviate from random allocations by incorporating and overtly discriminating based on a variety of patient features that should be ignored from a normative stance^12–19^. Although people disagree with discriminating policies^12^, judgments of concrete cases and abstract principles may diverge (e.g.^20^), explaining widespread evidence for discrimination in other work^13,14^. Beyond that, focusing on overt measures of discrimination may still underestimate the extent of biases and in-group favoritism due to implicit biases (i.e., biases that are not revealed when people are asked explicitly), social desirability, and under-reporting effects^21,22^. Thus, we move beyond assessing laypeople’s propensity for engaging in overt discrimination and study whether they reveal further biases by allowing them to use seemingly fair random allocations to discriminate covertly against less favored patients.

It is well-established that people’s judgments and decisions rarely adhere strictly to norms of moral reasoning^20,23–25^. People deviate from utilitarian norms of impartiality (e.g.,^20^) and egalitarian norms of equality^24^, and aim to avoid harming others, even if harming others would save a larger number of lives overall^23,25^. Laypeople also show a variety of pervasive biases, many of which align with their perceptions of how scarce medical resources should be allocated during crises (see^14^, for a review), and lead to systematic discrimination. Biases for health and longevity generally favor younger and healthier individuals, as shown in studies of QALY maximization (see^26^, for a review), survey experiments on the allocation of scarce medical resources^24,27^ and hypothetical triage scenarios during the COVID-19 pandemic^13–16,18,19,28^. Individuals are especially disfavored if they are perceived to be responsible for their ill-health, as in the case of obese individuals^29^ or with other medical conditions^13,18^ (but see^30^). People also tend to favor individuals who exhibit behaviors that are being perceived as ethical, are being described as cooperative and as contributing to society^31^. Similarly, patients who refrain from delinquent behaviors^13,16^, got vaccinated^32^, or abstained from risky (health) behavior^18^ are more likely to be favored. Relatedly, decision makers in triage decisions are willing to consider family-ties and caretaking roles, favoring patients with dependents^13,19^.

Individuals generally tend to cooperate more willingly with related others^33^ and with others that are perceived to belong to one’s in-group in economic games, such as the Prisoner’s dilemma or Dictator games^34–38^. These biases and in-group favoritism permeate into moral judgments and decisions and affect who would be saved in sacrificial dilemmas^24,39–44^. Such dilemmas include trolley-type dilemmas with self-driving cars^45^, but also triage situations involving the allocation of scarce healthcare resources^46^, where people also base their preferences on patients’ ethnic background^19^, citizenship^13,47^, whether patients recently migrated^48^, political affiliations (but see^49^), and shared nationality, especially when decision makers were more politically conservative^16^.

Manifestations of biases and favoritism need not be explicit and overt, but can remain implicit and operate in a covert fashion^50–52^, affecting both the offers in trust games^53^ and sacrificial dilemma decisions^54^. Related to modern forms of racism (e.g.,^55^), people supporting egalitarian norms more readily exhibit favoritism in situations that allow them to justify it, for instance, by punishing outgroup members more readily by rejecting unfair offers^56–58^. Moreover, people strategically use seemingly fair equality norms—such as randomness—in self-serving ways. They use random allocations to avoid responsibility for their outcomes^59^, and prefer the use of random devices to create plausible deniability^60^. Similarly, people use the alleged outcome of a coin flip to justify unequal allocations^61^ and judge equal allocations as less fair if an individual from their group was previously favored by an allocation decision^62^. Thus, people could use seemingly fair principles of random allocation to avoid responsibility, but also to justify biased decisions.

As a facet of avoiding responsibility, a bias for inaction that favors omission over commission^63,64^ has been linked to the avoidance of harm in sacrificial dilemmas^8,9,23^. In triage decisions, people are generally reluctant to withdraw care from patients who already receive treatment^12,15,19,32^. Effectively, this tendency manifests itself in a first-come, first-served principle, that is discouraged by ethical guidelines^1^. In contrast to this reluctance to withdraw, laypeople are more willing to harm a patient by withdrawing care from a patient with a worse medical prognosis^19^. Thus, deviations from norms of moral reasoning may be based on more general mechanisms of judgment and decision making, including in-group favoritism and omission bias, that may also affect the allocation of scarce medical resources.

Prior research on triage decisions primarily focused on explicit biases and overt discrimination. This focus may be prone to underestimate the true extent of biases and in-group favoritism^21,22^. Beyond replicating and extending the evidence for overt discrimination, we also examine covert discrimination tendencies. A diagnostic case for comparing covert and overt discrimination decisions is provided by situations in which a pre-allocated medical resource can be re-allocated. When withdrawing care causes active harm, strategically advocating random re-allocation provides an opportunity for covert discrimination that may appear to adhere to ethical guidelines. Rather than explicitly showing a bias by overtly reallocating a ventilator to a favored patient, a higher willingness to opt for random allocation when a ventilator has been pre-allocated to a disfavored patient than when it has been pre-allocated to a favored patient may superficially satisfy equality norms or ethical guidelines^1,10^. However, asymmetric use of random re-allocation would nonetheless violate impartiality by selectively increasing the survival chances of a favored patient, relative to keeping the ventilator pre-allocated to the unfavored patient. Thus, an asymmetric use of random re-allocation would provide an opportunity for covert discrimination, as it allows individuals to justify their choice and thus avoid blame by choosing a seemingly fair option^59^. Previous studies relying on forced choice experiments, conjoint analysis (estimating the relevance of multiple features from the responses to forced binary choices^16,18^), and ratings of moral relevance^13,48^, either did not allow individuals to explicitly abstain from a decision, or did not provide them with an opportunity for covert discrimination by avoiding direct harm.

The present study investigates whether laypersons advocate an overt allocation of scarce medical resources based on biases and in-group favoritism or covertly express their preferences through seemingly fair random re-allocation decisions. Participants decided for 19 patient pairs how a doctor should allocate the last remaining ventilator to one of two hypothetical patients during a viral pandemic. Since both patients were described to be in critical condition, saving one patient implied sacrificing the other patient. Each pair of patients differed only in one focal feature. As it was emphasized that patients did not differ in any other features (including medical prognosis), an impartial random ventilator allocation was the recommended normative response^1,10^. In contrast to most prior work on triage decisions, participants could always opt for random ventilator allocation (see^19^ for a similar approach). Using this setup enables us to disentangle two aspects of discrimination: first, comparing pairs of patients that differ in one distinctive feature allows for the assessment of interpersonal agreement on the bias towards favored vs. dis-favored patient characteristics. Second, stronger deviations from random allocations indicate more pervasive biases, whereas the extent of random allocations provides a measure of impartiality. To distinguish explicit biases in allocation decisions (by denying care to a disfavored patient) from implicit biases reflected in random re-allocation, we moved beyond the basic withholding condition (i.e., both patients arriving simultaneously), and included a withdrawal condition (i.e., one patient arriving before the other) at two time-points (3 and 4, out of a total of 6). In the withdrawal condition, the last ventilator had been pre-allocated to the patient who had arrived earlier, but this default could be revised by re-allocating it to the other patient or by opting for random re-allocation. This extends a similar study by Wilkinson et al.^19^, who also allowed for random allocation and used a withdrawal condition for patients with different survival probabilities. By varying the withdrawal condition for features that are orthogonal to patients’ survival probability, our study is the first to address covert discrimination in this setting. This novel approach to study covert discrimination reveals systematic and strong explicit biases towards the patients with features that were expected to be favored. This overt discrimination largely persists under withdrawal conditions, but we find no evidence for implicit biases revealed through the (mis-)use of randomness that could additionally harm disfavored individuals.

## Results and discussion

### Overt discrimination based on health and longevity, ethical behavior, family and caretaking, and demographics

**Figure 1:**
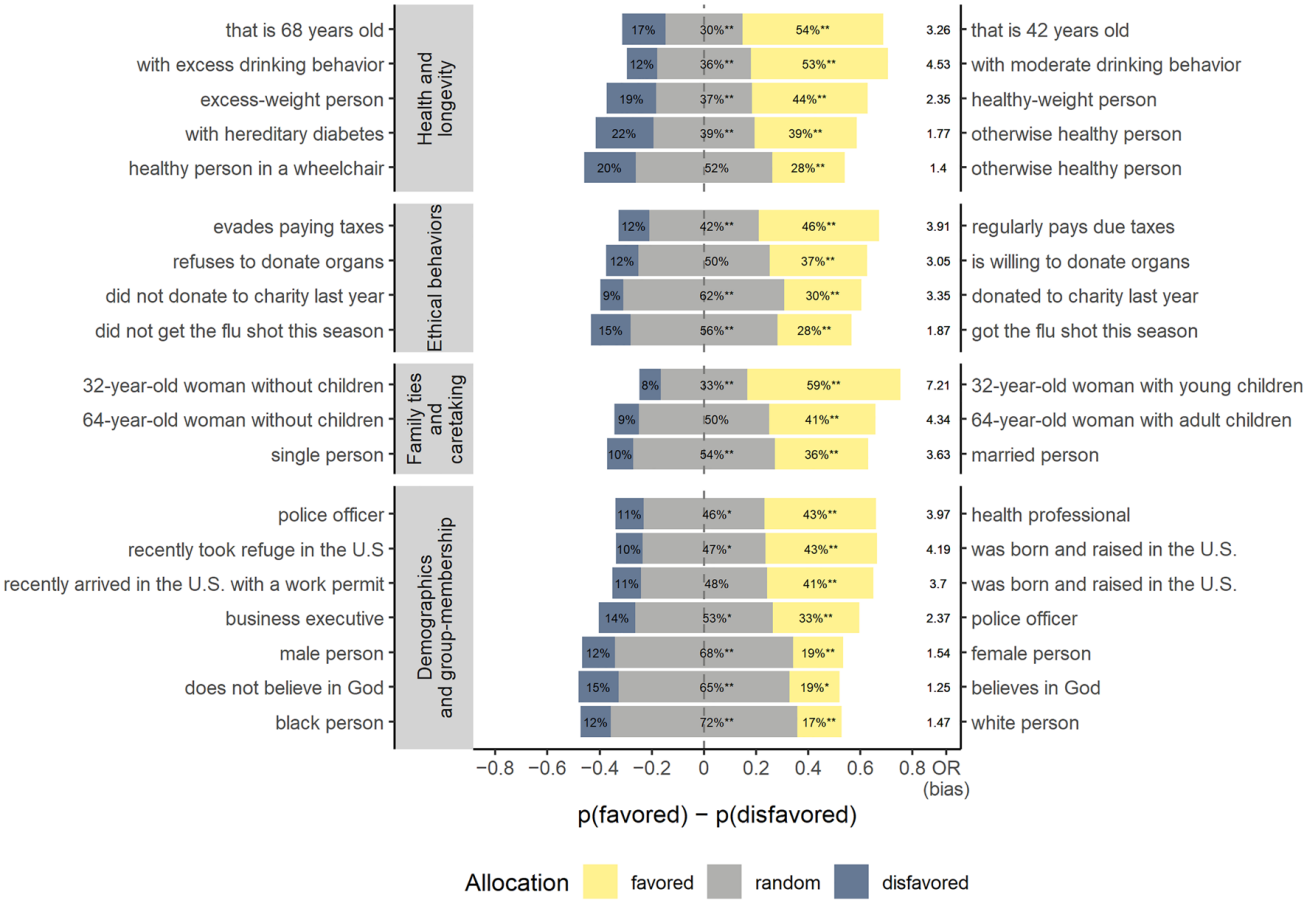
Discrimination reflected in proportions of random allocations vs. allocations to the favored and disfavored patient by patient feature. The right label corresponds to the patient who we predicted to be favored, and the left label to the patient who we predicted to be disfavored. All four feature clusters indicate that allocations varied between patient features. Asterisks on random allocations indicate that random allocations statistically deviated from 50%, reflecting a lack of impartiality, and asterisks on favored allocations indicate a higher likelihood for favored than for disfavored allocations among non-random allocations, reflecting systematic bias towards one patient (binomial tests against 50%, first asterisk uncorrected, second asterisk Bonferroni corrected for 19 tests). The offset of bars from the center indicate the degree of bias as the absolute difference between favored and disfavored allocations (in percent) and odds ratios (to the right of the bars) provide the effect size of each bias.

Across all features in the withholding condition (when both patients arrived simultaneously) we find evidence for feature-based discrimination on all 6 timepoints of data collection. Among the non-random allocations, we see strong agreement on a bias towards the patient with features that were expected to be favored [*p*(favored patient|random allocation) = 73*.*95%]. An average of 49*.*51% of nonrandom allocations across all features demonstrate a clear lack of an overall agreement on honoring impartiality. Thus, participants mostly agreed when expressing favoritism in the expected direction, but only a minority made consistently impartial allocation decisions (4.20% of participants always used random allocations, 7.20% of participants never did). At the same time, the extent of overt discrimination varied considerably between our 4 categories of patient features: health and longevity, ethical behavior, family and caretaking roles, and demographics. Figure 1 illustrates both the lack of impartiality (by random allocations, shown as grey bars, being substantially below 100%) and the biases towards favored patients (by more overt choices of favored features, shown in yellow, than of disfavored features, shown in blue) (results are largely robust to differences between time-points, see “Materials and methods” and Supplementary Sect. 2.1.3).

Figure 1 reveals interesting similarities and differences regarding the category and specific content of discriminating patient features (feature comparisons compare estimates of bias and impartiality from logistic mixed models and are controlled for all 6 timepoints of data collection, see Supplementary Table 4 for the models and Supplementary Sect. 2.1.3 for an exploration of an interaction with sets of time-points of data collection). Despite our emphasis that each pair of patients only differed in one central characteristic (i.e., not in their survival probability), participants showed particularly pervasive biases on most *health and longevity* features. Among features for which patient control is likely perceived to be low, there was a comparable amount of bias for a patient with hereditary diabetes and a patient in a wheelchair (*OR* = 0*.*96, *p* = 0*.*732), but impartiality towards the patient with diabetes was lower (*OR* = 0*.*45, *p* < 0*.*001). Comparing age to hereditary diabetes, in turn, shows both an increased bias (*OR* = 1*.*94, *p* < 0*.*001) and lower impartiality (*OR* = 0*.*55, *p* < 0*.*001) for age. This replicates earlier findings that patient age is used to discriminate in favor of a younger person^13–16,18,19,28^ (but see^30^; supplementary analyses also show no systematic evidence for ageism among younger participants, see Supplementary Sect. 2.3). When considering presumably more controllable features, excess drinking and excess weight elicited stronger biases than hereditary diabetes (*OR* = 3*.*07, *p* < 0*.*001 and *OR* = 1*.*46, *p* = 0*.*002 respectively), while bias against the excess-drinking patient was larger than against the excess-weight patient (*OR* = 2*.*11, *p* < 0*.*001). When contrasting the feature age with less controllable alcohol consumption, relatively lower bias for age (*OR* = 0*.*63, *p* < 0*.*001) could reflect the co-existence of general ageism with either prioritarian considerations for saving individuals in higher need^12^ or merit-based considerations in favor of the elderly^31^, that are absent for alcohol consumption. Conversely, decreased impartiality for age (*OR* = 0*.*66, *p* < 0*.*001) reflects the higher willingness to use age as a feature for these different reasons. Overall, these findings are in line with previous research findings that the elderly and individuals possibly responsible for their ill health (e.g., smokers, unvaccinated individuals, and those engaging in risky behaviors) were less likely to be saved^13–16,18^. Thus, perceptions of life-quality and individual control jointly play a role in resource allocation decisions.

With respect to *ethical behaviors*, we find consistent and clear biases towards patients that are being described as behaving more ethically (see Fig. 1). When patients differed in their charitable behavior and flu vaccination status, systematic choices of random allocations indicate that a majority of people honored impartiality in those cases (61.7% and 56.2%, respectively). However, this consensus on impartiality declined when one patient was described as an organ donor (50.4%) and further deteriorated when a patient was described to evade paying taxes (42.0%). Relative to the non-cooperative and delinquent case of tax evasion, the evidence for explicit biases based on other ethical features (e.g., vaccination and organ donation status) were weaker, presumably because they involve personal decisions without violating existing laws. Nevertheless, in-group effects (see Supplementary Sect. 2.3) suggest that those participants who reported the cooperative behavior themselves discriminated more in favor of the cooperative patient, possibly indicating instances of third-party punishment (e.g., like in the case of vaccination^32^).

Patient features implying *family-ties and caretaking roles* elicit substantive discrimination towards individuals who are embedded in family structures and occupy caretaking roles. The extent of biases increases and impartiality decreases from past caretaking to present caretaking. Regarding bias, we find no differences between married patients and those with adult children (*OR* = 1*.*03, *p* = 0*.*857), but increased biases towards patients with young children when compared to those with adult children (*OR* = 1*.*64, *p* = 0*.*001). However, participants became less impartial towards married patients than towards those with adult children (past caretaking, *OR* = 0*.*76, *p* = 0*.*003), and even less impartial towards patients with young rather than adult children (present caretaking, *OR* = 0*.*36*, p* < 0*.*001). Strong favoritism for saving a mother with young children corroborates previous findings that patients with dependents are more likely to be saved^13,65,66^. The decrease in impartiality from being married over past caretaking may indicate that contributing potential children in the future raises a patient’s perceived deservingness (e.g.,^13^), but not to the extent of already having contributed children in the past.

Finally, considering patients’ demographics and group membership, weak biases based on patients’ sex, ethnicity, and religiosity, and high impartiality on those features suggest that there were few signs of overt sexism or racism (but the observed biases can partly be related to in-group favoritism, see Supplementary Sect. 2.3). However, pronounced biases towards saving police officers and health professionals indicate favoritism for higher reputation jobs, thereby favoring health-professionals over police officers and business executives. Comparing health professionals to police officers shows a higher bias (*OR* = 1.86*, p* < 0.001) and lower impartiality (*OR* = 0.69, *p* < 0.001) for the former. This may reflect the instrumental value of healthcare workers during the pandemic, as reported in previous work^14,19,65^ and reflected in ethical guidelines^1,10^. With respect to residency status, there was substantive bias for patients born and raised in the U.S. over patients described as refugees or work permit holders, although both features lacked consensus on impartiality. Similarly, previous work reported favoritism towards citizens^13^, non-immigrants^47,48^, and patients of identical nationality^16,19^. If such expressions of favoritism were based on past rather than future contributions to society, we would expect work permit holders (who possibly could contribute soon) to be treated more favorably than refugees (who may not as easily contribute). However, neither was the bias for the individual born in the U.S. larger when compared to refugees rather than work permit holders (*OR* = 1.15, *p* = 0*.*348), nor was the corresponding impartiality decreased (*OR* = 0.93, *p* = 0*.*449). Participants’ reasoning could involve that those born and raised in the U.S. (or their parents) are already contributing to society (e.g., by paying taxes, see the role of tax evasion above). Alternatively, participants’ reasoning may reflect in-group favoritism or even racism that is openly shown, as it may appear to be justified based on perceived deservingness or past reciprocity^18,31^. In line with in-group favoritism, we find that impartiality was increased among more liberal participants, indicating that fewer of them discriminated (see Supplementary Sect. 2.3).

Although our pattern of results is largely unchanged upon in- or exclusion of the latter timepoints, minor differences (see Supplementary Sect. 2.3.1) may hint at changes in the participant pool or attitudes towards triage decisions over the course of the pandemic. That a trend towards lower bias holds across patient features could imply that all participants became less willing to rely on random allocations, which was largely driven by increased allocations to the unfavored patient (thus reducing overall bias). Moreover, some of the reported effects seem to be partly driven by participants’ group membership and thus reflect in-group favoritism (see Supplementary Sect. 2.3). Such in-group effects affected only the magnitude of bias or impartiality measures, but never reversed the direction of the bias among participants sharing the features of the unfavored patient (with the exception of non-religious individuals, who exhibited a bias against religious patients). Thus, even when incorporating in-group favoritism, participants’ discrimination patterns are driven by overarching preferences for the favored patient.

### Overt but no covert discrimination: No sign of asymmetric re-allocation through randomness

Given clear explicit biases on all of our patient features and a wide-spread willingness to deviate from impartial resource allocation, we now turn to potential implicit biases, as operationalized by the withdrawal conditions (in which the last ventilator had been pre-allocated by default to either a favored or unfavored patient who arrived earlier).

**Figure 2:**
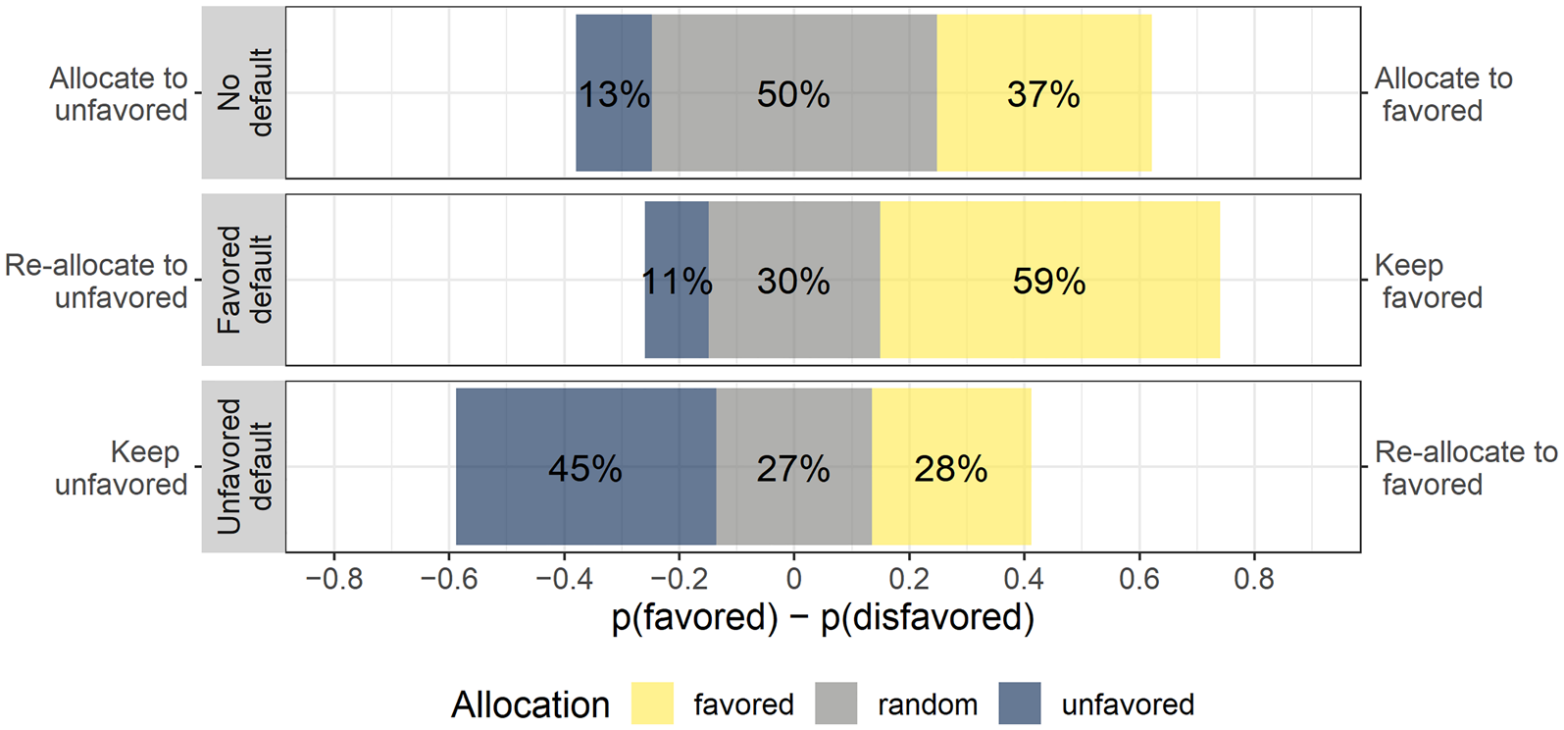
Probability of receiving the ventilator by condition across patient features. Probabilities to allocate the ventilator to either patients in withholding scenarios (top) vs. retaining or re-allocating it in a withdrawal scenario in which the ventilator has been pre-allocated to a favored default (middle) or to an unfavored default (bottom).

Figure 2 illustrates the willingness for allocating the ventilator in the withholding condition versus keeping it allocated or re-allocating it in the two possible withdrawal conditions. Overall, the allocation probability differed between conditions (according to a logistic mixed model with random effects for participants and patient features, *χ*^2^(2) = 167*.*858*, p* < 0*.*001). The default patient was more likely to keep the ventilator than it was allocated to the favored patient in the withholding condition for both, favored or unfavored defaults (*OR* = 4*.*77*, p* < 0*.*001 and *OR* = 1*.*63*, p* < 0*.*001, respectively). This shows a strong default effect. A higher probability of keeping the ventilator for the favored default than for the unfavored default (*OR* = 2*.*93*, p* < 0*.*001), indicates that the favoritism observed in situations of withholding generalized to situations of withdrawal. This extends the previously documented effects regarding resource withdrawal based on differential survival probability^19^ and whether a patient was unvaccinated against COVID-19^32^.

Selectively opting for more random re-allocations for less favored patients provided participants with an opportunity for covert discrimination. Although we find an overall effect indicating a difference in random re-allocations between the withdrawal and withholding conditions [*χ*^2^(2) = 399*.*920*, p* < 0*.*001], participants’ choices for seemingly impartial random re-allocations were not asymmetrically more likely for disfavored defaults (*OR* = 1*.*17, *p* = 0*.*562, with more random re-allocations towards the favored default). The overall effect merely reflects fewer random re-allocations relative to those in the withholding condition for both favored (*OR* = 0*.*15*, p* < 0*.*001) and unfavored defaults (*OR* = 0*.*13*, p* < 0*.*001). Consequently, we find no evidence that people systematically used egalitarian random allocations to rationalize their preference against an unfavored default by asymmetrically increasing seemingly fair random allocations, as reported for other settings^59,61^. Instead of engaging in covert discrimination, many participants appeared to use a first-come first-served rule, but still endorsed overt discrimination against the unfavored patient (with the collapsed pattern being robust for all patient features, and for different times of data collection, see Supplementary Sects. 2.2.1 and 2.2.3).

## General discussion

Across a variety of different patient features, we investigated how laypeople decided to withhold and withdraw care in the context of triage decisions. Replicating and extending previous work. participants’ withholding decisions showed overt discrimination. They predictably favored more healthy patients and patients with higher status jobs, like health professionals and police officers, but also prioritized patients who are socially embedded and in care-taking roles, as well as patients showing cooperative behaviors. Surprisingly, overt discrimination persisted in withdrawal situations in which a life-saving ventilator could be re-allocated. Rather than using random allocations to covertly express implicit biases, participants consistently made overt re-allocation decisions. Overall, pervasive explicit biases based on patient features profoundly shaped laypersons’ triage decisions, even in situations where the decision concerned the withdrawal of care.

The pervasiveness of discrimination adds to a body of findings that laypersons do not strictly adhere to ethical guidelines^13–16,19,32,65,66^ that focus on patient prognosis^2,11^ and otherwise recommend random resource allocation^1,10^. Analogous to judgments about whether social welfare is deserved^31^, laypeople consider patients as deserving care to different degrees, based on their perceived level of control over the situation, but also based on societal contributions. Our results on excess weight and alcohol consumption add to mixed findings from conjoint experiments which either find^18^ or do not find^30^ evidence for these (seemingly) controllable features, but agree on the importance of different professions. Likewise, our findings on patients who recently migrated, but also on marriage, adult children, and young dependents could also reflect a judgment of deservingness due to past or future contributions to society. Interestingly, participants here incorporated features that they had indicated to be irrelevant when asked to judge their moral relevance^13^. They also discriminated based on patients’ social usefulness, although they largely rejected discriminating triage policies^12^. This likely implies potential differences between judgements of abstract policies and concrete hypothetical decisions^20^, which could partially consist in opportunities to rationalize discrimination based on features of the specific situation. Additional analyses also show that allocation decisions also reflect the pervasive phenomenon of in-group favoritism^34–38^. The relevance of in-group favoritism for triage decisions has so far only been shown for nationality^16^ and cooperative behaviors, such as getting vaccinated^32^. Strikingly, discrimination persisted in situations in which individuals had to decide about actively harming a patient by re-allocating a critical resource. However, people did not seize the opportunity to rationalize their favoritism through seemingly fair random allocations in withdrawal conditions. This is surprising for two reasons: first, people reliably seem reluctant to withdraw care in triage scenarios^12,15,19,32^, avoid directly harming others in sacrificial dilemmas^8,9,23,25^, and exhibit a general preference for inaction over action^63,64^. Second, the observed absence of covert discrimination is at odds with previous reports of the motivated use of randomness^59–61^ and people’s use of different norms of fairness on outgroup individuals^56,57^.

We cannot fully exclude that experimenter demand characteristics due to salient features or a general aversion against randomness^67^ may have led to an overestimation of the degree of discrimination, whereas for some features random allocations may have been more likely due to social desirability (e.g., ethnicity and sex). However, demand effects have been found to be lower in online experiments than in laboratory settings^68^. A general avoidance of random allocations also conflicts with the fact that random allocations were typically the modal response in situations of withholding. Additionally, our discrimination patterns persisted in the withdrawal condition where there was no individual accountability for covert discrimination, which would reduce the potential impact of social desirability. Thus, even though the estimated probabilities of discrimination may be overestimated due to demand characteristics or randomness aversion, the rank order of features should be unaffected and reveal actual preferences. Participants’ proneness to openly show their favoritism, even in situations of withdrawal extends the mixed evidence that people are more willing to reallocate a resource from a patient unvaccinated against COVID-19^32^ or with a lower survival probability^15,19^. In sum, discrimination appears to be pervasive and extends beyond features that can be aligned with ethical guidelines, like health-related outcomes and a person’s instrumental value in conditions of scarcity.

Overall, investigations of hypothetical triage scenarios offer a valuable window into human moral reasoning, which may be subject to a variety of moral motives^43^. The willingness to engage in trade-offs between patient features and situational aspects is in line with broadly utilitarian cost–benefit calculations^69^, but violates norms of impartiality^20^ and deontologic rules against harm^20,25,70^. Future work should aim to improve our understanding of the conditions under which people rely on overt and covert forms of discrimination. Although here we were interested in comparing isolated features to get a clean estimate of which features are used, investigating patients described with feature combinations could reveal additional insights, such as trade-offs or interactions between features. This would also be beneficial for external validity, because typically patients will vary in several features. Our results on single features add valuable insights to laypeople’s perspectives on practical debates about triage scenarios^14^. Rather than directly translating into guidelines, participants’ willingness to overtly express biases and in-group favoritism provides a cautionary note: Results reveal that there are substantial discrepancies between ethical guidelines and people’s moral intuitions, resulting in challenging disagreements over particular decisions. At the same time, the fact that people’s biases are openly stated rather than covertly enacted enables policy makers to anticipate such discrepancies, allowing—but also requiring—them to better explain expert recommendations to the general public.

## Materials and methods

### Participants

In total, we recruited 2463 participants on Amazon Mechanical Turk (39.1% female, mean age of 37.2 years, ranging from 18 to 83 years; see Supplementary Table 1 for a full sample description). We collected data on 6 time-points in 1-week intervals between March 2nd and May 23rd and on August 27th and September 3rd, 2020. Data were always collected during U.S. working hours (beginning from about 8 a.m. to about 2 p.m. and lasting no later than 5 p.m.) in order to control for possible effects of weekday and time of day on the participant pool^71^. Participants provided informed consent prior to participating in the study. Checks of plausibility and completion time, as well as self-reports of data quality were used to screen out participants who did not pay sufficient attention. We excluded the data from a total of 167 participants due to missing data, missed attention checks, or text-field entries suggesting automated responses. This resulted in a total sample of *N* = 2296 participants (or about 1362 participants in the withholding condition across timepoints, plus about 460 participants in each withdrawal condition at timepoints 3 and 4). The study adhered to the Declaration of Helsinki, relevant laws, and institutional guidelines, as certified by IRB of the University of Konstanz. The University of Konstanz’s IRB approved the study. All participants provided informed consent.

### Design

Participants made a total of 19 hypothetical decisions to which of two patients a doctor should allocate the only available ventilator during a viral epidemic that had created a scarcity of medical resources. They could either decide to allocate the ventilator to one of the two patients or decide to use a randomization device (like a random lottery draw or fair coin flip). The two patients in each of the scenarios were described to differ in exactly one focal feature (implying no difference in survival probability). This allowed us to address the question how different patient features affect participants’ allocation decisions, without introducing noise through interaction effects or potential diversions of richer representations. Based on our expectations of who will be favored due to their feature (e.g., based on higher contributions to society) we distinguish between favored and disfavored patients in the following. For a list of patient pairs see Fig. 1 (pairs are aligned so that the individual we expected to be saved with a higher probability is placed on the right). The instructions clearly stated that the patient who received the ventilator would be saved, whereas the other patient would die. In time-points 3 and 4 we additionally introduced a withdrawal condition and varied between-subjects whether the favored patient, the disfavored patient, or neither patient had been pre-allocated the last available ventilator. Thus, the decision became whether to retain the default allocation, withdraw and re-allocate the ventilator to the other patient, or opt for re-allocation by means of a randomization device. An example scenario can be found in the supplement (see Supplementary Fig. 1).

### Procedure

Participants first provided informed consent, received instructions that they will be asked to make decisions about which one of two comparable patients differing in one key regard should receive life-saving treatment. Patient pairs were presented in a randomized order. Next, participants answered questions about their own risk and their severity perceptions of the current COVID-19 crisis, their own health, altruism, time and risk preferences, as well as demographic questions, including their state and county, their political views and religiosity. To allow for the detection of in-group effects, we asked participants to provide information on characteristics equal or related to the characteristics of our hypothetical patients (e.g., political orientation and religious beliefs). Moreover, they provided information on their county so that we were able to assess the objective severity of the COVID-19 crisis in their area of residence. Participants always had the option to leave questions blank.

### Data analyses

Our two main outcomes were (a) the proportion of random allocations, with lower proportions indicating less impartiality patient feature, and (b) the proportion of allocations to the patient whom we expected to be favored among non-random allocations, with higher proportions indicating a bias towards the corresponding patient feature. Unless an analysis explicitly refers to a withdrawal condition, analyses are based on the withholding condition. None of the analyses were pre-registered. To compare allocation probabilities within patient features, we used binomial tests against 50% on either allocation alternative. In order to control for variation between participants and patient features, all other analyses used pairs of logistic mixed models (using lme4) for allocations to the patient who was expected to be favored (or keeping, see below) among non-random allocations (denoted by *P(favored|non-random)* in the formulas below) and random allocations. For allocations to favored patients, we included the proportion of non-random allocations as an additional predictor. Across these models, we report Wald-*χ*^2^ tests (indicating the relevance of predictors) and calculated comparisons of interest from post hoc tests (using emmeans), correcting for multiple tests with a Sidak correction. All discrete variables were effect-coded and all continuous variables were *z*-standardized, with the exceptions of age, which was mean-centered. The first pair of models compared allocation patterns between patient features with fixed effects for patient features and time point of data collection, including a random effect for the participant, capturing unexplained variance between individuals (e.g., though differences in randomness aversion).

Model of bias (including the probability of random responses for participants and features):

$$\begin{gathered} P\left( {favored|non - random} \right) \sim b_{feature} + b_{timepoint} + p\left( {non - {\text{ r}}andom|participant} \right) \, \hfill \\ + p\left( {non - random|feature} \right) \, + \sigma_{participant} \hfill \\ \end{gathered}$$

Model of impartiality:

$$P\left( {random} \right) \, \sim b_{feature} + b_{timepoint} + \sigma_{participant}$$

Models describing our default conditions included random effects for both patient features and participants. The second pair of models compared the conditions of withholding and withdrawal for random allocations and the probability that the patient who already received care could keep the ventilator, including fixed effects for condition (withholding, withdrawing from favored, withdrawing from unfavored) and timepoint of data collection. Since situations of withholding lacked a patient receiving care, we used the probability that the expected to be favored patient received the ventilator as a comparison benchmark.

Model of keeping the ventilator:

$$P\left( {keep} \right) \, \sim b_{default \, condition} + b_{timepoints} + \sigma_{participant} + \sigma_{feature}$$

Model of random allocations in situations of withdrawal:

$$P\left( {random} \right) \, \sim b_{default \, condition} + b_{timepoints} + \sigma_{participant} + \sigma_{feature} .$$

## Supplementary Information

Supplementary Information.

## Data Availability

All data, code, and materials have been made publicly available at OSF and can be accessed at https://osf.io/pn239/.
