# Supplementary material for: Explicit discrimination and ingroup favoritism, but no implicit biases in hypothetical triage decisions during COVID-19
Source: Sci Rep. 2024 Jan 12;14:1213. doi: 10.1038/s41598-023-50385-w (PMC10786932; doi:10.1038/s41598-023-50385-w)
Supplement: Supplementary file 1 — Supplementary Information. [file 41598_2023_50385_MOESM1_ESM.pdf]

Supplementary materials:
Explicit discrimination and ingroup favoritism, but no implicit biases in hypothetical triage decisions during COVID-19

Nico Gradwohl[1,2,3,*], Hansjörg Neth[1,2], Helge Giese[1,2,4], and Wolfgang Gaissmaier[1,2]

[1]Department of Psychology, University of Konstanz, Konstanz, Germany
[2]Centre for the Advanced Study of Collective Behaviour, University of Konstanz, Konstanz, Germany
[3]Department for the Ecology of Animal Societies, Max Planck Institute of Animal Behaviour, Konstanz, Germany
[4]Charité – Universitätsmedizin Berlin, Berlin, Germany
[*]nico.gradwohl@uni-konstanz,de

Contents

1 Supplementary methods **2**
1.1 Example scenarios 2
1.2 Supplementary Measures 2
1.3 Detailed sample description 3
1.4 Statistical models 6
2 Supplementary results **7**
2.1 Overt discrimination based on health and longevity, ethical behavior, family and caretaking, and demographics 7
Full test statistics for bias and impartiality on patient features • Description of patient features • Robustness to timepoint of data collection
2.2 Overt but no covert discrimination: no sign of asymmetric re-allocation through randomness 12
Robustness to patient features • Model table for random allocations and the probaiblity that the default keeps the ventilator • Robustness of overt instead of covert discrimination to timepoint of data collection
2.3 Feature-specific differences in overt discrimination effects by participant characteristics indicate in-group favoritism . . 14
References **21**

1 Supplementary methods

1.1 Example scenarios

Task 1

A viral epidemic has spread across the globe infecting and killing thousands of people. The number of severely infected people surpasses the capacity limits at the local hospital.

At the moment, there are **two patients** whose survival depends on immediately receiving artificial ventilation, but there is only **one ventilator** available.

A doctor is in charge of assigning patients to the available ventilators.

The two patients are comparable in every aspect, except for **their profession**.

One person will live, the other will die. **To whom** should the doctor assign the only ventilator available?

**Assign the ventilator to the police officer.**	**Use a randomization process (such as a lottery) to assign the ventilator.**	**Assign the ventilator to the health professional.**
○	○	○

Task 1

A viral epidemic has spread across the globe infecting and killing thousands of people. The number of severely infected people surpasses the capacity limits at the local hospital.

At the moment, there are **two patients** whose survival depends on immediately receiving artificial ventilation, but there is only **one ventilator** available.

A doctor is in charge of assigning patients to the available ventilators.

The two patients are comparable in every aspect, except for **their religious beliefs** and the fact that

- **the person who believes in God** arrived earlier today and is currently kept alive by the only ventilator available, while
- **the person who does not believe in God** just arrived at the hospital and will die when not receiving ventilation soon.

Thus, the doctor must decide whether to withdraw the ventilator from the person who believes in God in order to keep the person who does not believe in God alive.

One person will live, the other will die. **How** should the doctor assign the only ventilator available?

**Keep the ventilator assigned to the person who believes in God.**	**Use a randomization process (such as a lottery) to assign the ventilator.**	**Reassign the ventilator to the person who does not believe in God.**
○	○	○

Supplementary Figure 1. Example scenarios as seen by the participants in the baseline condition without a default (upper panel) and with a default (lower panel).

1.2 Supplementary Measures

To address in-group favoritism we collected participant characteristics that were related to our patient features. Political views and religiosity were assessed with simple items using a 7-point scale (from conservative to liberal) and non-religious to religious as used by[1]. We asked about alcohol consumption as standard drinks per day[2]. In our analysis, we contrasted moderate alcohol consumption (0 to 2 standard drinks per day) with hazardous alcohol consumption (more than 2 standard drinks per day). To assess participants' weight, we asked them to provide their height and weight (to calculate their BMI) and excluded participants

with implausible entries. Finally, we asked participants whether they are registered as an organ donor, whether they got the seasonal flu shot, and assessed their attitude towards flu vaccination on a 5-point scale (1: strongly oppose to 5: strongly favor).

Additional questions regarding time- and risk-preferences, as well as altruistic giving were simple 1-item measures taken from the preference survey module[3,4]. To assess altruism, participants indicated how much of US-$1,000 they are willing to spend on a good cause. Finally, we also assessed participants' perceptions of the COVID pandemic. We used 7-point scales translated into English from Betsch et al.[5] asking for the probability (1: extremely unlikely to 7: extremely likely) and severity of an infection (1: extremely harmless to 7: extremely severe), as well as the perceived severity of the COVID-19 outbreak in their area of residence (1: completely harmless to 7: very severe).

1.3 Detailed sample description

Supplementary Table 1 describes the collected participant characteristics for the sample used in our analyses (for the sample without exclusions see Supplementary Table 2.

At the latter 2 timepoints of data collection (5 and 6) the sample tended to report higher education, higher belief in god, having more children, higher income, more likely were African American, being more likely married, being less likely born in the US, were more likely under- or normal-weight, more likely reported excellent health, but also COVID-infections. Additionally, they more likely selected the riskier of two options and donated more in a hypothetical donation task.

A change in sample composition is consistent with the observation that the participant pool on Amazon Mechanical Turk has changed during the pandemic[6]. To account for this possible change we enter timepoint of data collection into our models and show our main descriptive results also by different ranges for our timepoints of data collection.

Supplementary Table 1. Description of participant characteristics for the sample with exclusions at each timepoint of data collection.

	Timepoint of data collection					
	1 (n = 229)	2 (n = 231)	3 (n = 708)	4 (n = 684)	5 (n = 227)	6 (n = 217)
**Condition**						
non-preferred default	0	0	231	237	0	0
no default	229	231	234	224	227	217
preferred default	0	0	243	223	0	0
**Demographics**						
Female	44.5	44.2	40.7	37.1	35.2	38.2
Age	37.7	38.6	37.3	37.6	35.9	35.7
Religiosity	3.2	3.5	3.7	3.6	4.9	5.0
Political	4.6	4.8	4.6	4.7	4.3	4.4
**Any children**						
Any children	35.8	44.2	46.5	43.6	60.4	73.7
**Education**						
none	0.4	0.4	0.1	0.1	0.0	0.0
high school	24.9	20.3	15.8	17.1	7.5	7.4
post high	13.5	12.1	10.5	10.4	4.4	2.8
vocational	2.6	3.5	2.8	2.5	1.8	0.5
BA	48.5	44.2	52.1	51.2	58.6	62.2
MA	9.6	16.5	16.4	17.0	27.3	26.3
PhD	0.4	3.0	2.3	1.8	0.4	0.9
**Income (in US-$)**						
$\leq$ 5000	3.1	6.9	3.5	5.7	3.1	3.7
$<$ 5000	5.7	4.3	4.5	4.5	3.1	1.8
$<$ 10000	13.5	8.7	12.9	12.9	5.3	7.4
$<$ 15000	9.6	8.7	8.1	9.8	10.1	8.8
$<$ 25000	16.6	13	13.1	12.7	14.1	12
$<$ 35000	23.1	16.9	19.4	19.2	22.5	30.4
$<$ 50000	21.8	34.6	30.6	25.7	34.4	25.8
$<$ 80000	6.6	6.9	7.9	9.5	7.5	10.1
**Ethnicity**						
White/Caucasian	78.2	80.1	70.1	74.4	67.8	70.5
African American	9.2	10.4	15.5	12.6	24.7	22.1
Asian American	7.0	4.3	6.6	6.4	3.5	4.6
American Indian or Alaskan Native	0.4	0.9	0.7	1.0	1.3	0.0
Native Hawaiian or Pacific Islander	0.0	0.0	0.1	0.3	0.0	0.0
Latino	3.5	2.6	5.1	3.9	1.8	1.8
Other	1.7	1.7	1.8	1.3	0.9	0.9
**Marital status**						
single	43.7	38.1	39.0	37.1	25.1	18.4
partner	15.7	13.4	9.7	12.4	7.5	7.4

married	40.6	48.5	51.3	50.4	67.4	74.2
not say	0.0	0.0	0.0	0.0	0.0	0.0
**Time in the U.S.**						
less than 20 years	7.0	8.7	12.0	8.5	15.0	21.7
migrated recently	4.8	4.8	6.2	7.3	9.7	7.4
born in the U.S.	88.2	86.6	81.8	84.2	75.3	71.0
**Residence area (population)**						
village (< 3000)	4.8	6.5	5.4	4.5	4.4	6.5
small town (3000 to 15,000)	14.0	11.7	16.0	14.9	15.0	17.5
town (15,000 to 100,000)	27.9	32.9	31.6	33.3	28.6	25.3
city (100,000 to 1,000,000)	35.8	33.3	31.6	27.8	36.6	35.0
large city (> 1,000,000)	17.5	15.2	15.0	18.6	14.5	15.2
don't know	0.0	0.4	0.4	0.9	0.9	0.5
**Standard drinks**						
none	46.3	35.9	32.5	36.7	22.0	25.3
1-2	33.6	39.8	39.0	36.1	37.9	36.9
3-4	13.1	18.6	18.1	17.1	26.4	25.3
5-6	6.6	3.9	7.5	6.7	9.3	9.7
7-9	0.4	0.9	2.0	2.2	2.6	1.8
10+	0.0	0.9	1.0	1.2	1.8	0.9
**BMI**						
0 to 18.5	6.1	8.2	12.9	11.1	33.9	36.9
18.5 to 25	39.3	45.5	44.4	42.8	37.4	39.6
25 to 30	32.3	29.0	28.1	29.4	18.5	13.8
> 30	22.3	17.3	14.7	16.7	10.1	9.7
**Own health**						
excellent	27.9	27.7	27.1	23.2	33.0	34.1
good	50.7	57.1	60.0	63.5	59.0	55.8
fair	20.5	13.4	11.6	11.7	7.9	8.8
poor	0.9	1.7	1.3	1.6	0.0	1.4
**Own COVID infection**						
yes	2.2	3.5	5.1	3.4	11.5	14.3
not confirmed	1.7	2.6	3.5	3.7	8.4	11.5
no	87.8	89.6	86.3	86.3	75.8	70.5
can't tell	8.3	4.3	5.1	6.7	4.4	3.7
**Someone with COVID known**						
yes	6.1	10.0	13.0	12.7	20.3	30.0
not confirmed	3.5	6.5	8.6	7.3	15.9	15.7
no	86.0	79.2	74.4	75.1	59.0	51.2
can't tell	4.4	4.3	4.0	4.8	4.8	3.2
**Vaccination**						
Flu vaccination attitude	3.8	3.9	3.7	3.8	3.7	3.8
Flu vaccination	59.0	58.9	58.3	59.6	54.2	46.5
**Organ donation**						
Organ donation	54.6	55.0	54.1	59.1	65.2	62.7
**Decision preferences**						
Larger later	27.9	34.6	36.6	35.1	30.4	34.6
Risky option	11.4	14.3	16.2	16.5	24.7	33.2
Altruism (amount of 1000)	139.7	173.8	203	195.4	328.5	341

Supplementary Table 2. Description of survey information for the sample without exclusions at each timepoint of data collection.

	Timepoint of data collection					
	1 (n = 248)	2 (n = 250)	3 (n = 751)	4 (n = 728)	5 (n = 247)	6 (n = 239)
**Condition**						
non-preferred default	0	0	247	247	0	0
no default	248	250	245	239	247	239
preferred default	0	0	259	242	0	0
**Demographics**						
Female	43.1	43.6	39.8	37.1	36.8	36.8
Age	37.2	38.1	37.1	37.7	35.9	36.1
Religiosity	320.6	352.8	378.2	359.9	482.6	496.2
Political	462.1	478.8	454.6	464.0	433.6	435.1
**Any children**						
Any children	34.7	45.2	47.2	44.7	60.2	73.6
**Education**						
none	0.4	0.4	0.1	0.1	0.0	0.0
high school	24.6	18.8	15.2	16.5	6.9	7.9
post high	14.9	11.6	10.3	10.0	5.3	2.9
vocational	2.4	4.0	2.8	2.7	2.4	0.4
BA	47.6	45.6	52.2	51.6	57.1	61.9
MA	9.7	16.8	17.2	17.3	27.9	25.9
PhD	0.4	2.8	2.1	1.6	0.4	0.8
**Income (in US-$)**						
$\leq$ 5000	2.8	6.9	3.5	5.9	2.8	3.8
$<$ 5000	6.1	4	4.5	4.4	3.6	2.1
$<$ 10000	13.4	9.3	12.7	12.3	5.7	7.1
$<$ 15000	10.1	9.3	8.3	10.2	10.5	9.7
$<$ 25000	15.8	13.3	13.2	12.6	13	11.8
$<$ 35000	23.5	16.9	19.3	19	21.1	30.3
$<$ 50000	22.3	33.5	30.7	26.1	36	25.6
$<$ 80000	6.1	6.9	7.9	9.5	7.3	9.7
**Ethnicity**						
White/Caucasian	78.6	80.0	69.1	73.8	66.8	68.6
African American	9.3	10.4	16.1	13.2	25.5	23.8
Asian American	6.5	4.8	6.9	6.6	4.0	4.2
American Indian or Alaskan Native	0.4	0.8	0.7	1.1	1.2	0.0
Native Hawaiian or Pacific Islander	0.0	0.0	0.3	0.3	0.0	0.0
Latino	3.2	2.4	5.1	3.8	1.6	2.5
Other	2.0	1.6	1.9	1.2	0.8	0.8
**Marital status**						
single	45.6	37.2	38.6	36.4	26.1	18.6
partner	15.7	13.2	9.5	12.6	7.8	7.2
married	38.7	49.2	51.1	49.9	65.7	72.6
not say	0.0	0.4	0.8	1.1	0.4	1.7
**Time in the U.S.**						
less than 20 years	6.9	8.8	13.4	9.2	15.4	23.3
migrated recently	4.4	5.2	6.1	7.3	9.3	7.2
born in the U.S.	88.7	85.9	80.4	83.5	75.2	69.5
**Residence area (population)**						
village (< 3000)	5.7	6.4	5.3	5.0	4.5	5.9
small town (3000 to 15,000)	13.8	11.6	16.2	15.5	15.0	18.4
town (15,000 to 100,000)	29.1	32.9	31.4	32.5	29.6	26.4
city (100,000 to 1,000,000)	34.0	33.3	31.2	27.9	35.6	33.9
large city (> 1,000,000)	17.4	14.5	15.3	18.0	14.6	15.1
don't know	0.0	1.2	0.5	1.1	0.8	0.4
**Standard drinks**						
none	46.4	35.2	33.2	36.9	22.3	25.1
1-2	32.3	40.4	37.5	35.8	38.9	36.0
3-4	13.7	18.4	18.4	16.9	25.5	25.9
5-6	6.9	4.0	7.9	7.2	8.9	10.0
7-9	0.8	0.8	2.1	2.1	2.8	2.1
10+	0.0	1.2	0.9	1.2	1.6	0.8
**BMI**						
0 to 18.5	6.1	9.2	13.0	12.6	33.6	37.7
18.5 to 25	38.4	44.6	44.0	42.5	37.3	39.7
25 to 30	33.5	29.3	28.6	28.6	19.3	13.4
$>$ 30	22.0	16.9	14.3	16.4	9.8	9.2

**Own health**						
excellent	27.8	28.8	27.4	23.6	32.2	33.5
good	52.0	57.2	60.1	63.3	58.8	56.9
fair	19.0	12.4	11.3	11.4	9.0	8.4
poor	1.2	1.6	1.2	1.7	0.0	1.3
**Own COVID infection**						
yes	2.8	3.6	6.1	4.3	12.6	14.3
not confirmed	1.6	2.4	4.0	4.0	7.7	12.2
no	86.3	90.0	85.0	85.0	75.3	69.7
can't tell	9.3	4.0	4.9	6.7	4.5	3.8
**Someone with COVID known**						
yes	6.5	10.4	13.7	13.3	20.2	29.3
not confirmed	3.6	6.4	8.5	7.8	16.6	16.3
no	85.1	79.2	73.8	74.2	58.3	51.0
can't tell	4.8	4.0	4.0	4.7	4.9	3.3
**Vaccination**						
Flu vaccination attitude	3.8	3.9	3.7	3.8	3.7	3.8
Flu vaccination	60.1	59.2	57.3	59.2	53.8	44.8
**Organ donation**						
Organ donation	52.4	54.8	54.2	58.8	65.2	62.3
**Decision preferences**						
Larger later	29.8	34.4	36.2	35.0	31.6	36.0
Risky option	12.1	14.4	17.8	16.8	24.3	33.9
Altruism (amount of 1000)	140.2	177.8	203.8	198.2	319	336.9

1.4 Statistical models

Here, we describe the models used in our analyses. They include the logistic mixed models to analyze differences between features, differences between conditions of withholding and withdrawal across features, and feature-specific effects beyond the other features. We relied on mixed models whenever we had to account for non-independence of observations.

All analyses were conducted in R-4.1.2[7]. Generalized mixed effects models were estimated using the function `glmer` in `lme4`[8]. We omitted random effects that could not be estimated based on the available data.

We specified theoretically plausible models and tested the relevance of the corresponding predictors and their interactions. To test main effects and interactions we used the R-package `car`[9]. For fixed effects in logistic models (including logistic mixed models) we provide Wald-χ^2 statistics and their corresponding p-values[10]. These tests allow to test the relevance of variables in logistic models[11], including predictors with more than 2 levels[12], similar to omnibus-tests in an ANOVA framework. For comparisons between features we report odds ratios and p-values from *post hoc* tests obtained through[13].

Feature specific models To assess feature-specific effects we report model pairs for combinations of patient feature and decision maker characteristic, on both bias and impartiality. Effects are only feature-specific if an interaction of the patient feature with the participant characteristic explains that data better than the main effects of patient feature and participant characteristic. Otherwise, they rather reflect the general effects of the patient feature or participants general response tendencies, respectively. Therefore, models included an indicator for the focal patient feature, the decision maker characteristic, and their interaction. Since timepoints of data collection 1 to 4 differ from timepoints 5 and 6, we include an interaction for these 2 sets of timepoints and discuss robustness of our results. To address non-independence of features and individuals we include random intercepts for both. For models of bias we also include the proportion of random allocations for the respective participant and feature.

For both bias and impartiality we report Wald χ^2 statistics to qualify whether the 2-way interactions of patient feature and participant characteristic are relevant for the model. For discrete participant characteristics we report odds ratios across the remaining patient features, odds ratios for the focal patient feature and whether their differences differ from each other (OR_{diff}). For continuous characteristics, we report the difference in effects between the focal and the remaining features.

Additionally, we test 3-way interactions of patient feature, participant characteristic and set of timepoints to assess if the effects differ between timepoint 1 to 4 and timepoint 5 and 6. If the interaction is relevant for the model, we report the subsequent comparisons by set of timepoints.

$$
\begin{aligned}
& bias \sim b_{feature} + b_{characteristic} + b_{timepoint} + \\
& b_{feature} \times b_{characteristic} + b_{feature} \times b_{timepoint} + \times b_{characteristic} \times b_{timepoint} + b_{feature} \times b_{characteristic} \times b_{timepoint} + \\
& p(non-random|individual) + p(non-random|feature) + \sigma_{participant} + \sigma_{feature}
\end{aligned} \tag{1}
$$

$$
\begin{aligned}
& impartiality \sim b_{feature} + b_{characteristic} + b_{timepoint} + \\
& b_{feature} \times b_{characteristic} + b_{feature} \times b_{timepoint} + \times b_{characteristic} \times b_{timepoint} + b_{feature} \times b_{characteristic} \times b_{timepoint} + \\
& \sigma_{participant} + \sigma_{feature}
\end{aligned} \tag{2}
$$

Supplementary Tables 6 to 11 show the models tested.

2 Supplementary results

2.1 Overt discrimination based on health and longevity, ethical behavior, family and caretaking, and demographics

2.1.1 Full test statistics for bias and impartiality on patient features

See next page.

Supplementary Table 3. Probability of random allocation and favored among non-random allocations (with p-values for difference to 0.5) for each patient pair. The total number of allocations was N=1362.

Patients differ by (feature)	Disfavored patient	Favored patient	*n*(non-random)	p(favored|non-random)	p(random)
Health and longevity					
age	that is 68 years old	that is 42 years old	958	0.76 (0.000)	0.30 (0.000)
alcohol consumption	with excess drinking behavior	with moderate drinking behavior	874	0.82 (0.000)	0.36 (0.000)
physical fitness	excess-weight person	healthy-weight person	862	0.70 (0.000)	0.37 (0.000)
health diabetes	with hereditary diabetes	otherwise healthy person	833	0.64 (0.000)	0.39 (0.000)
health wheelchair	healthy person in a wheelchair	otherwise healthy person	651	0.58 (0.000)	0.52 (0.110)
Ethical behaviors					
financial honesty	evades paying taxes	regularly pays due taxes	790	0.80 (0.000)	0.42 (0.000)
organ donor status	refuses to donate organs	is willing to donate organs	676	0.75 (0.000)	0.50 (0.807)
charity-related behavior	did not donate to charity last year	donated to charity last year	522	0.77 (0.000)	0.62 (0.000)
vaccination behavior	did not get the flu shot this season	got the flu shot this season	596	0.65 (0.000)	0.56 (0.000)
children 32	32-year-old woman without children	32-year-old woman with young children	911	0.88 (0.000)	0.33 (0.000)
Family ties and care-taking					
children 64	64-year-old woman without children	64-year-old woman with adult children	683	0.81 (0.000)	0.50 (0.935)
relationship status	single person	married person	621	0.78 (0.000)	0.54 (0.001)
profession health	police officer	health professional	730	0.80 (0.000)	0.46 (0.009)
residence permit status	recently took refuge in the U.S	was born and raised in the U.S.	721	0.81 (0.000)	0.47 (0.032)
living in the U.S	recently arrived in the U.S. with a work permit	was born and raised in the U.S.	705	0.79 (0.000)	0.48 (0.203)
Demographics and group-membership					
profession police	business executive	police officer	644	0.70 (0.000)	0.53 (0.048)
sex	male person	female person	430	0.61 (0.000)	0.68 (0.000)
religious beliefs	does not believe in God	believes in God	470	0.56 (0.019)	0.66 (0.000)
ethnicity	black person	white person	388	0.59 (0.000)	0.71 (0.000)

2.1.2 Description of patient features

Supplementary Table 4. Estimates from a logistic mixed model predicting the probability of (1) favored allocations among non-random allocations (bias) and (2) random allocations (impartiality) as a function of patient feature, timepoint, controlling for the number of non-random allocations on the participant and feature level in model 1.

	Dependent variable:			
	Bias: p(favored | nonrandom)		Impartiality: p(random)	
	(1)		(2)	
Constant	1.669***	(0.055)	−0.062	(0.049)
Feature: alcohol consumption	0.367***	(0.095)	−0.837***	(0.066)
Feature: charity-related behavior	0.409***	(0.111)	0.736***	(0.066)
Feature: children 32	0.900***	(0.107)	−1.012***	(0.067)
Feature: children 64	0.406***	(0.105)	0.016	(0.064)
Feature: ethnicity	−0.299**	(0.112)	1.402***	(0.071)
Feature: financial honesty	0.259**	(0.096)	−0.454***	(0.064)
Feature: health diabetes	−0.755***	(0.083)	−0.648***	(0.065)
Feature: health wheelchair	−0.713***	(0.089)	0.156*	(0.064)
Feature: living in the U.S	0.239*	(0.100)	−0.080	(0.064)
Feature: organ donor status	0.135	(0.097)	0.047	(0.064)
Feature: physical fitness	−0.378***	(0.084)	−0.781***	(0.066)
Feature: profession health	0.396***	(0.099)	−0.190**	(0.064)
Feature: profession police	−0.226*	(0.096)	0.187**	(0.064)
Feature: relationship status	0.378***	(0.105)	0.288***	(0.064)
Feature: religious beliefs	−0.734***	(0.102)	0.983***	(0.067)
Feature: residence permit status	0.376***	(0.102)	−0.150*	(0.064)
Feature: sex	−0.300**	(0.107)	1.182***	(0.069)
Feature: vaccination behavior	−0.367***	(0.095)	0.399***	(0.064)
Timepoint 2	0.295**	(0.106)	0.450***	(0.108)
Timepoint 3	−0.006	(0.098)	0.049	(0.107)
Timepoint 4	0.173+	(0.104)	0.306**	(0.110)
Timepoint 5	−0.414***	(0.094)	−0.650***	(0.110)
Timepoint 6	−0.563***	(0.095)	−0.590***	(0.112)
p(non-random|individual)	−0.128***	(0.019)		
Number of Individuals	1298		1362	
$\sigma_{participant}$	1.215		1.688	
AIC	13318.51		27987.87	
Nakagawa's-R^2	0.10		0.11	
LR-χ^2	574.42***		2132.41***	
Note:			*p<0.1; **p<0.05; ***p<0.01	

2.1.3 Robustness to timepoint of data collection

Supplementary Figure 2 shows that allocation patterns across features differed between the first 4 and the last 2 timepoints of data collection. Therefore, we investigate the impact of these two time windows on our results. Overall we find that the general pattern of results is robust to timepoint of data collection (see Supplementary Figure 3). Despite decreased random allocations, the direction of bias remains the same across timepoints. Still, overall it appears that bias is decreased for several features through more allocations to the unfavored patient. Next we discuss the robustness of the results reported in the main text.

Health and longevity For all *health and longevity* features bias is decreased in the last 2 timepoints of data collection. Most remarkably biases based on the diabetes feature and the wheelchair feature largely disappear. Except for the comparison between the hereditary diabetes feature and the wheelchair feature, the pattern of all comparisons reported in the main text remains unchanged (although some effects fail to reach conventional levels of statistical significance).

Comparing patients differing in the presence of hereditary diabetes and patients differing whether they sit in a wheelchair, absent evidence for bias is robust from the first 4 timepoints ($OR = 1.02$, $p = 0.903$) to the last 2 timepoints ($OR = 0.86$, $p = 0.440$). Similarly, the direction of effects is the same as across timepoints with lower impartiality for the diabetes features ($OR = 0.31$, $p < 0.001$) but no discernible difference in the last 2 timepoints ($OR = 0.95$, $p = 0.735$). Thus, perceptions of

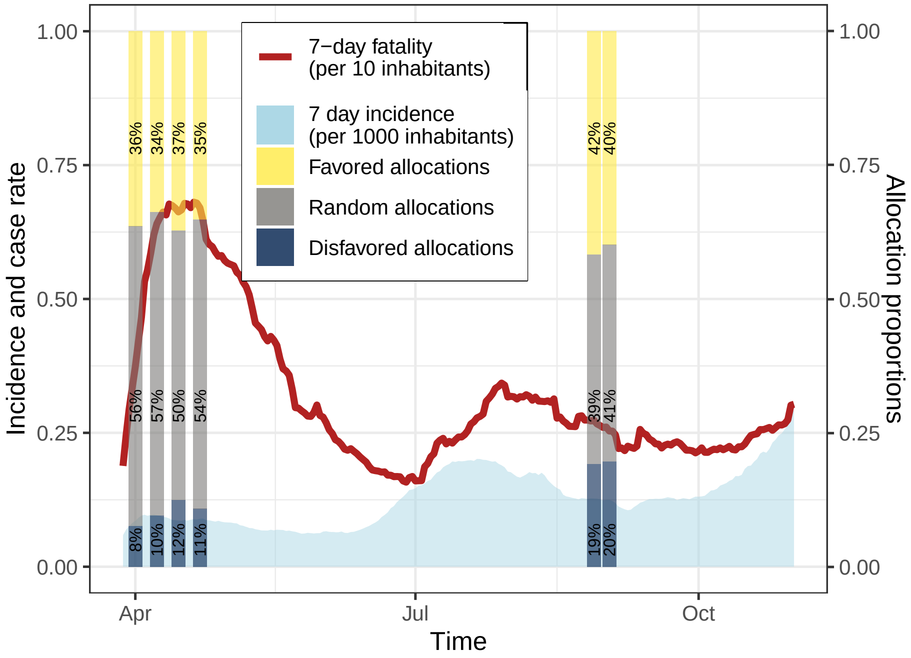

Supplementary Figure 2. Allocations at each timepoint of data collection next to COVID-19 cases, deaths, and 7-day incidence (and events) around these timepoints. We observe no clear preference for random allocations, but systematic partiality in favor of the patient we expected to be favored. Moreover, our data show a systematic decrease in random allocations indicating higher relevance of features and a decrease in partiality, indicating less strong favoritism for the overall favored patient.

different kinds of illness or frailty may differ based on the surveyed participants or temporal changes in attitudes. The change in effects appears mainly driven by changes in the wheelchair feature (Supplementary Figure 3).

Comparing alcohol consumption and diabetes, increased bias is robust (timepoints 1 to 4: $OR = 3.09$, $p < 0.001$ and timepoints 5 and 6: $OR = 3.16$, $p < 0.001$ respectively). Likewise, the direction of effects is robust when comparing excess weight and diabetes (timepoints 1 to 4: $OR = 1.57$, $p = 0.004$ and timepoints 5 and 6: $OR = 1.36$, $p = 0.096$ respectively), with an even stronger bias against the excess drinking individual in the first 4 ($OR = 1.97$, $p < 0.001$) and last 2 timepoints ($OR = 2.32$, $p < 0.001$).

Similarly, comparing patient age and diabetes, the effect sizes differ, whereas the patterns for bias (timepoints 1 to 4: $OR = 1.66$, $p = 0.001$ and timepoints 5 and 6: $OR = 2.46$, $p < 0.001$) and impartiality (timepoints 1 to 4: $OR = 0.50$, $p < 0.001$ and timepoints 5 and 6: $OR = 0.67$, $p = 0.020$ respectively) are robust. Finally, contrasting patient age and alcohol consumption, decreased bias is largely limited to the first 4 timepoints ($OR = 0.54$, $p < 0.001$, despite an effect in the same direction in timepoints 5 and 6: $OR = 0.78$, $p = 0.210$). Correspondingly, the decreased impartiality on age relative to alcohol consumption is clearly limited to timepoints 1 to 4 ($OR = 0.57$, $p < 0.001$, timepoints 5 and 6: $OR = 0.93$, $p = 0.660$). Thus, the age and alcohol consumption features were treated more similarly to each other over time. Overall, these robustness checks underscore that controllable features play a substantive role for participants' allocation decisions, whereas considerations of frailty may vary with situational factors or sample characteristics.

Ethical behaviors When patients features differed in their charitable behavior and flu vaccination status, systematic choices of random allocations reflecting impartiality are limited to timepoints 1 to 4 (68.6% and 63.5%, respectively) but not in the last 2 timepoints (charitable behavior: 47.3%) with a reversed preference for non-random allocations for vaccination behavior 41.2%). Similarly, organ donor status elicited fewer random responses than than charitable behavior and vaccination in the first 4 timepoints (55.6%) and even fewer in the latter 2 (39.6%). The latter two findings could imply an increase in polarization on the topics of vaccination and organ donation or an increase in selecting response options randomly, resulting in a more even distribution of the 3 response categories. The clear decline in impartiality relative to the other features when one patient was described to evade taxes persisted from the first 4 timepoints (45.9%) to the last 2 timepoints (34.0%), attesting to the robustness of pervasive punishment of non-cooperative or even delinquent behavior.

Family ties and caretaking Favoritism for those with *family ties* persists but is weakened in later timepoints of data collection. Participants were less impartial towards married patients than towards those with adult children (past caretaking) in timepoints 1

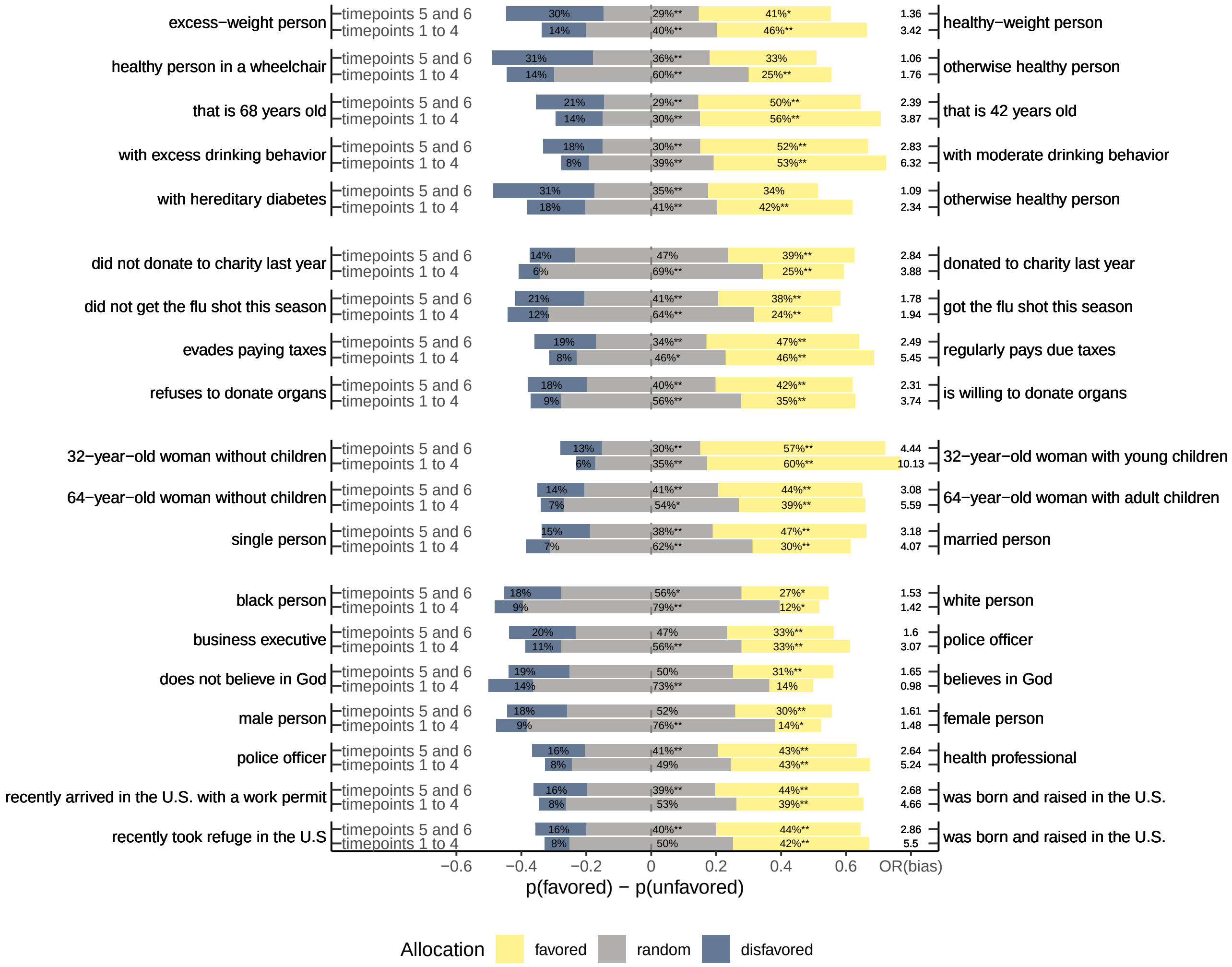

Supplementary Figure 3. Allocation proportions by patient feature across timepoints of data collection. The left label corresponds to the patient who we expected to be disfavored and the right label to the patient who we expected to be favored. Asterisks indicate that random allocations significantly deviated from 50%, and that were biased towards the favored patient, respectively (binomial tests against 50%, first uncorrected, second Bonferroni corrected). The offset of bars indicates bias the as the absolute difference between favored and disfavored allocations in percent and odds ratios right of the bars provide effect sizes of bias

to 4 ($OR = 0.60$, $p < 0.001$) but not in timepoints 5 and 6 ($OR = 1.22$, $p = 0.212$). Participants still were less impartial towards patients with young rather than adult children (present caretaking, (timepoints 1 to 4 $OR = 0.31$, $p < 0.001$ and timepoints 5 and 6 $OR = 0.50$, $p < 0.001$). We still do not find differences in bias between married patients and those with adult children (timepoints 1 to 4: $OR = 1.19$, $p = 0.414$ and timepoints 5 and 6: $OR = 0.89$, $p = 0.598$) and the pattern increased bias towards patients with young children when compared to those with adult children is persistent (timepoints 1 to 4: $OR = 1.77$, $p = 0.008$), despite a marginal effect on timepoints 5 and 6 ($OR = 1.50$, $p = 0.072$). Thus, our results concerning family ties appear largely robust, especially the favoritism towards a mother with young children. However, it is not entirely clear if patients' relationship status is actually weaker than other indicators of family, like having adult children.

Demographics and group membership The pattern of effects of patients' *demographics and group membership* is largely robust, although the results of demographic features are less clear at timepoints 5 and 6. Although random allocations overall decreased, impartiality on patients' sex, ethnicity and religiosity remained larger than on all other features in the last 2 timepoints of data collection (see Figure 3). However, whereas model-based comparisons of bias on each of these features with the average of bias across all remaining features, reveal smaller than average bias in timepoints 1 to 4 (patient sex: $OR = 0.59$, $p = 0.001$, patient ethnicity:, $OR = 0.60$, $p = 0.002$, patient religiosity: $OR = 0.26$, $p < 0.001$) there was no conclusive evidence for this difference in the last 2 timepoints, despite the direction remaining the same (patient sex: $OR = 0.89$, $p = 0.465$, patient ethnicity:, $OR = 0.87$, $p = 0.363$, patient religiosity: $OR = 0.83$, $p = 0.238$). Conversely, model-based comparisons of impartiality reveal smaller than average impartiality in both, timepoints 1 to 4 (patient sex: $OR = 4.70$, $p < 0.001$, patient ethnicity:, $OR = 5.91$, $p < 0.001$, patient religiosity: $OR = 3.47$, $p < 0.001$) and timepoints 5 and 6 (patient sex: $OR = 2.13$, $p < 0.001$, patient ethnicity:, $OR = 2.67$, $p < 0.001$, patient religiosity: $OR = 1.96$, $p < 0.001$). The decrease in impartiality and the decrease in differences in bias might reflect more random selection of the response option, increasing the selection of both the favored and unfavored patients at the expense of fewer random allocations.

Favoritism for higher reputation jobs is robust, since comparing health professionals to police officers at both the first 4 and the last 2 timepoints shows evidence for a higher bias ($OR = 1.91$, $p = 0.001$ and $OR = 1.81$, $p = 0.005$) and lower impartiality ($OR = 0.67$, $p < 0.001$ and $OR = 0.72$, $p = 0.040$). This may indicate that higher reputation jobs robustly elicit favoritism.

Finally, bias towards the patient born and raised in the U.S. over patients described as refugees or work permit holders is decreased but persists (see Figure 3). We still do not find evidence for increased bias for the individual born in the U.S. when compared to refugees rather than work permit holders for either set of timepoints ($OR = 1.21$, $p = 0.334$ and $OR = 1.08$, $p = 0.730$), as well as decreased impartiality ($OR = 0.88$, $p = 0.274$ and $OR = 1.04$, $p = 0.803$). Although larger effects at timepoints 1 to 4 seem more in line with the expectation that work permit holders may be treated more favorably because they can contribute more readily to society, we have to reject this conjecture in across our current data.

Summary Overall, our results appear to be robust to the time of data collection. Nevertheless, evidence for some effects is limited to the first 4 timepoints of data collection. One possible explanation is that the discriminatory power of patient features was lower in the later timepoints. In line with this explanation, results on strong features like tax evasion and young children were changed less, whereas the results for several other features are blurred. The decreased discriminatory power could be due to changes in the participant pool (see Supplementary Table 1, also documented by[6].

First, increased similarity of the 3 categories could indicate less careful responding in the last 2 timepoints, reflecting more random responding (which would reflect 33% of allocations in each category). The decrease in impartiality and the decrease in differences in bias might reflect more random selection of the response option, increasing the selection of both the favored and unfavored patients at the expense of fewer random allocations. Alternatively, the dynamics of the COVID-19 pandemic could have genuinely altered participants' decision behavior, because they became more familiar with making such decisions over the course of the pandemic. For instance, increased polarization could have been increased through an increased willingness to engage in allocations, also towards the initially less favored patient.

Our data do not allow to properly disentangle these competing explanations. Crucially, our main conclusions about the general reliance on most patient features and our experimental investigation of situations of withdrawal are robust to the differences between timepoints of data collection. Future work should aim to clarify the role of realism and public discourse for this type of decision task in longitudinal data, excluding differences in the participant pool.

2.2 Overt but no covert discrimination: no sign of asymmetric re-allocation through randomness

2.2.1 Robustness to patient features

Supplementary Figure 4 illustrates that the pattern of results was robust for all patient features: Random allocations were never larger when the default is the unfavored individual and the favored patient typically kept the ventilator more likely than the unfavored patient.

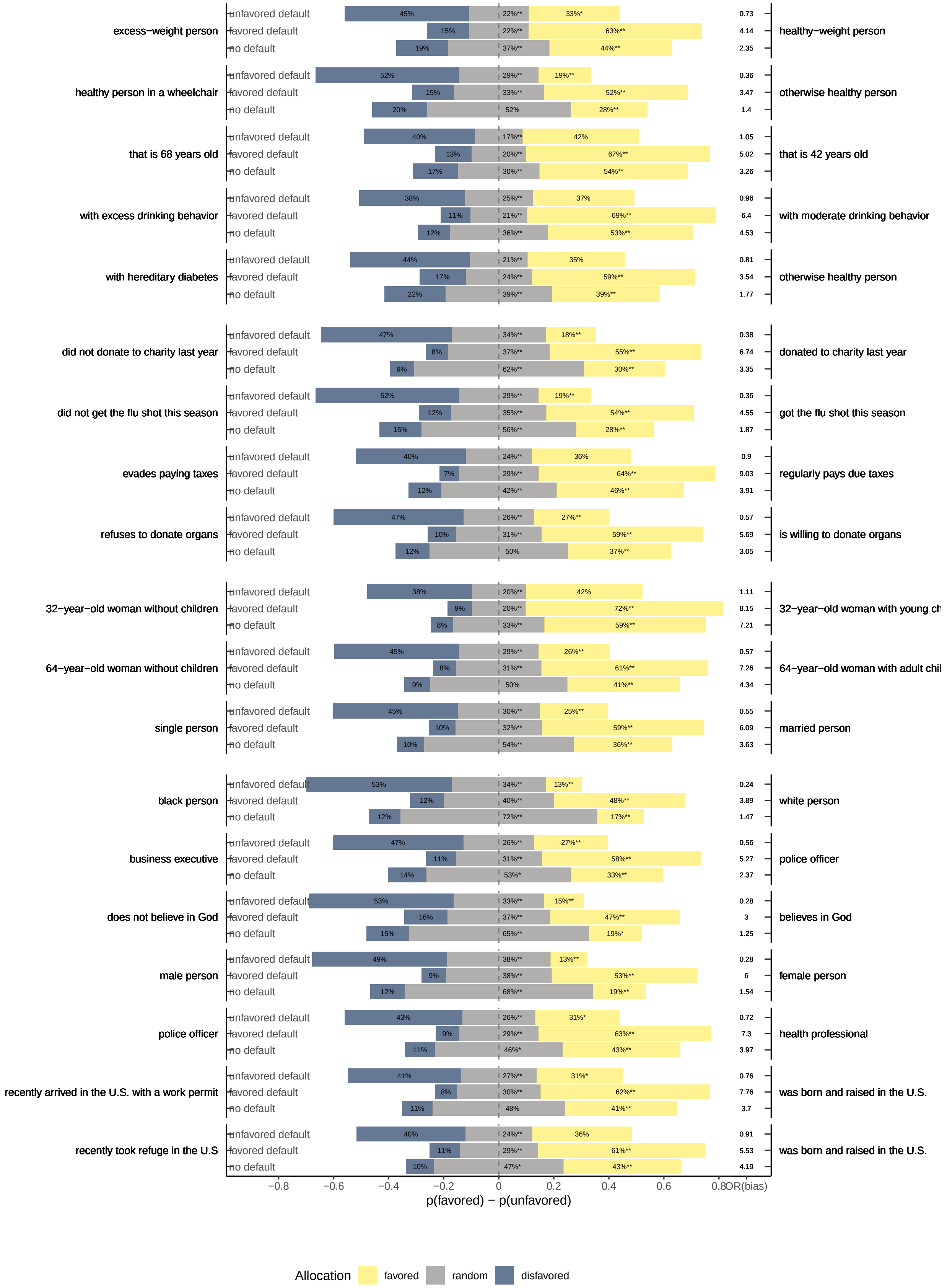

Supplementary Figure 4. Probability that the favored patient receives or keeps the ventilator as a function of who has it for each feature.

2.2.2 Model table for random allocations and the probaiblity that the default keeps the ventilator

Supplementary Table 5. Estimates from a logistic mixed model predicting the probability of (1) random allocations and (2) whether a patient keeps the ventilator when one of the patients arrived earlier (default). If there was no default (reference condition) keeping was defined as the favored patient receiving the ventilator. Results are controlled for whether the timepoint of data collection was among the first 4 or not.

	Dependent variable:			
	Relevance: p(random)		Keeping: p(keep)	
	(1)		(2)	
Constant	-1.635^{***}	(0.164)	0.039	(0.120)
Favored default	-0.618^{***}	(0.083)	0.894^{***}	(0.069)
Disfavored default	-0.779^{***}	(0.082)	-0.178^{**}	(0.068)
Later 2 timepoints	-0.487^{***}	(0.064)	0.159^{**}	(0.053)
$\sigma_{participant}$	2.094	1.745		
$\sigma_{feature}$	0.639	0.453		
AIC	42494.42		46902.42	
Nakagawa's-R^2	0.10		0.05	
LR-χ^2	385.01^{***}		225.76^{***}	
N	43624		43624	
n_{ind}	2296		2296	
Note:	$^{+}$ p < 0.1; * p < 0.05;** p < 0.01; *** p < 0.001.			

2.2.3 Robustness of overt instead of covert discrimination to timepoint of data collection

Comparisons of the default conditions to the witholding condition in the first 4 and last 2 timepoints reveal that our results are robust to differences in participant behavior. For both earlier and later timepoints, the probability that the favored default could keep the ventilator was consistently larger compared to allocating it to the favored patient in situations of withholding ($OR = 5.01$, $p < 0.001$ and $OR = 3.64$, $p < 0.001$). The probability that the unfavored default kept the ventilator was larger than allocating it to the favored patient in the withholding condition in timepoints 1 to 4 ($OR = 1.71$, $p < 0.001$) without evidence for the effect in timepoints 5 and 6 ($OR = 1.25$, $p = 0.301$). For both timepoints random allocations were less likely for both the favored ($OR = 0.13$, $p < 0.001$ and $OR = 0.35$, $p < 0.001$) and unfavored patient ($OR = 0.11$, $p < 0.001$ and $OR = 0.30$, $p < 0.001$). Differences at the last 2 timepoints were weaker, because the bias towards the favored individual was smaller and random allocations were generally more rare. Note that comparisons between favored and unfavored default patients were not affected by the last 2 timepoints of data collection (5 and 6) because the experimental manipulation was limited to timepoints 3 and 4, so that we do not know if responses to situations of withdrawing care would have been different in later timepoints.

2.3 Feature-specific differences in overt discrimination effects by participant characteristics indicate in-group favoritism

Beyond main effects of patient features we find evidence for interactions of patient features with participant characteristics, reflecting in-group favoritism and decision maker ideology (see Supplementary Figure 5). We distinguish feature specific differences in partiality and bias between participants from response tendencies that are general to patient features or participant characteristics by means of logistic mixed models. By testing the interaction between the feature and the decision maker interaction, we show the effect that a combination of a patient feature and its corresponding participant characteristic have beyond the underlying tendencies (see Supplementary Methods; for model tables see Supplementary Tables 6 to 12). For absent differences in bias, differences in impartiality still indicate in-group favoritism, because a larger proportion of favored individuals receives the ventilator.

Age Supplementary Table 6 shows the feature specific model for patient and participant age. There is no evidence for an interaction between participant age and the age feature for bias [$\chi^2(1) = 0.028$, $p = 0.868$] or impartiality [$\chi^2(1) = 0.493$, $p = 0.483$]. Absent evidence for moderating influence of timepoint of data collection on bias [$\chi^2(1) = 0.003$, $p = 0.956$] or impartiality [$\chi^2(1) = 1.983$, $p = 0.159$] indicates robustness of this finding. Thus, seemingly lower bias in favor of the younger patient among younger participants in Supplementary Figure 5 does not appear to be specific to patient age and reflects the absence of additional ageism.

Organ donation For organ donation we find evidence for more willingness to discriminate among participants who were registered as organ donors themselves (Supplementary Table 6). Although we do not find evidence for a feature specific effect on bias [$\chi^2(1) = 2.247$, $p = 0.134$, robust to later timepoints $\chi^2(1) = 0.145$, $p = 0.703$], there is evidence for feature-specific differences in impartiality as a function of whether participants were registered as organ donors or not [$\chi^2(1) = 26.543$, $p <$

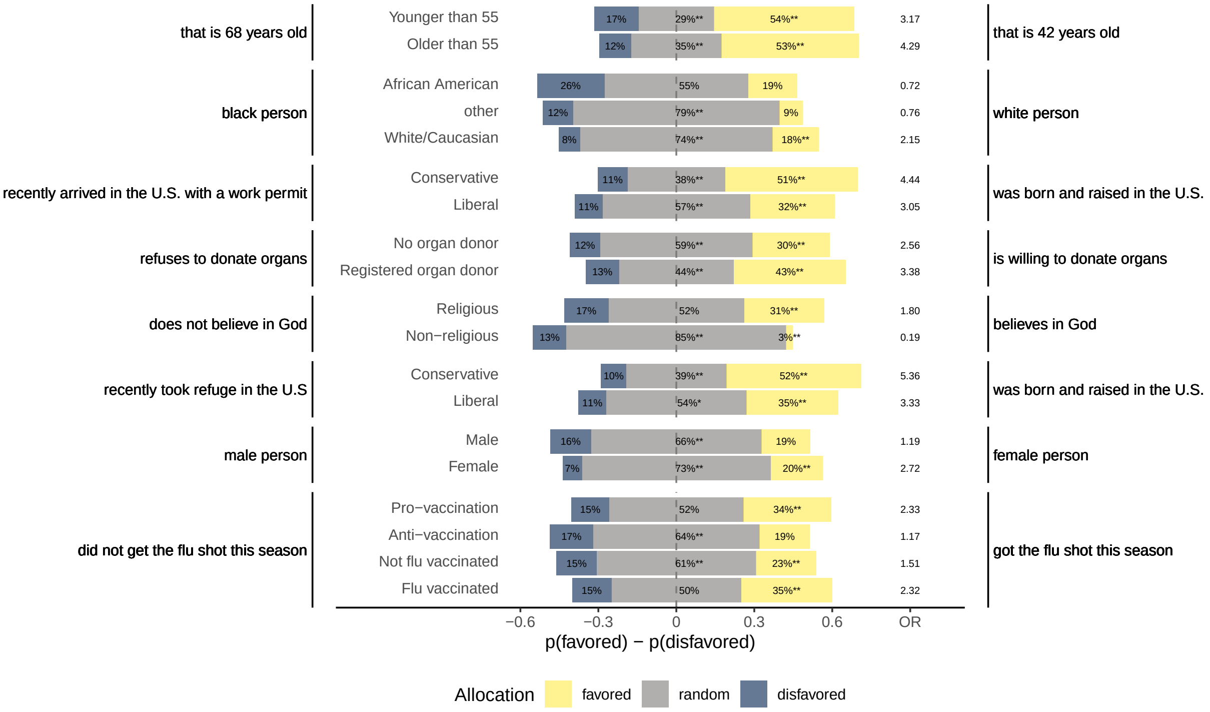

Supplementary Figure 5. Feature specific allocation patterns by participant groups corresponding to patient features. We observe in-group favoritism for a large number of patient features. The left label corresponds to the patient who we expected to be disfavored and the right label to the patient who we expected to be favored. Asterisks indicate that random allocations significantly deviated from 50%, and that favored allocations were significantly more likely that disfavored allocations, respectively (binomial tests against 50%, first uncorrected, second Bonferroni corrected). The offset of bars indicates bias the as the absolute difference between favored and disfavored allocations in percent and odds ratios right of the bars provide effect sizes of partiality.

Supplementary Table 6. Estimates from a logistic mixed model predicting allocations to the young patient as a function of participants' age. Model (1) predicts allocations to the the young patient among non-random allocations (bias) and Model (2) random allocations.

	Dependent variable:			
	Bias: p(favored | nonrandom)		Impartiality: $p(random)$	
	(1)		(2)	
Constant	0.852	(0.807)	-0.727^{*}	(0.341)
p(non-random|individual)	-0.125^{***}	(0.018)		
p(non-random|feature)	0.192^{+}	(0.115)		
Feature: Age	−0.200	(0.232)	-0.577^{+}	(0.337)
Age (mean-centered)	0.013^{*}	(0.006)	0.019^{**}	(0.006)
Last 2 timepoints	-0.323^{***}	(0.060)	-0.244^{***}	(0.063)
Feature: Age × Age (mean-centered)	0.001	(0.004)	0.003	(0.004)
Feature: Age × Last 2 time points	0.030	(0.045)	0.223^{***}	(0.039)
Age (mean-centered) × Last 2 time points	−0.001	(0.006)	−0.005	(0.006)
Feature: Age × Age (mean-centered) × Last 2 time points	0.000	(0.004)	−0.005	(0.004)
$\sigma_{participant}$	1.203		1.684	
$\sigma_{feature}$	0.402		0.651	
AIC	13376.25		28023.12	
Nakagawa's-R^2	0.06		0.05	
$LR\text{-}\chi^2$	69.77^{***}		129.46^{***}	
N	13065		25878	
n_{ind}	1298		1362	
Note:	$^{+}p < 0.1$; $^{*}p < 0.05$; $^{**}p < 0.01$; $^{***}p < 0.001$.			

Supplementary Table 7. Estimates from a logistic mixed model predicting allocations to the organ donor as a function of whether participants reported being registered as organ donors. Model (1) predicts allocations to the the organ donor among non-random allocations (bias) and Model (2) random allocations.

	Dependent variable:			
	Bias: p(favored ∣ nonrandom)		Impartiality: $p(random)$	
	(1)		(2)	
Constant	1.335*	(0.678)	−0.163	(0.374)
p(non-random∣individual)	−0.127***	(0.018)		
p(non-random∣feature)	0.151	(0.107)		
Feature: Organ donation	0.059	(0.216)	0.045	(0.371)
Registered organ donor	0.094	(0.066)	−0.196**	(0.063)
Last 2 timepoints	−0.350***	(0.067)	−0.453***	(0.063)
Feature: Organ donation × Registered organ donor	0.078	(0.052)	−0.191***	(0.037)
Feature: Organ donation × Last 2 timepoints	−0.004	(0.052)	−0.013	(0.037)
Registered organ donor × Last 2 timepoints	−0.096	(0.066)	−0.049	(0.063)
Feature: Organ donation × Registered organ donor × Last 2 timepoints	−0.020	(0.052)	0.077*	(0.037)
$\sigma_{participant}$	1.212		1.693	
$\sigma_{feature}$	0.409		0.714	
AIC	13380.66		28026.26	
Nakagawa's-R^2	0.06		0.03	
$LR\text{-}\chi^2$	65.36***		126.31***	
N	13065		25878	
n_{ind}	1298		1362	
Note:	$^+$p < 0.1; *p < 0.05;**p < 0.01; ***p < 0.001.			

Supplementary Table 8. Estimates from a logistic mixed model predicting allocations to the patient who was vaccinated against the flu as a function of participants' attitude towards flu vaccination and whether participants reported being vaccinated against the flu. Model (1) predicts allocations to the the patient who was vaccinated against the flu among non-random allocations (bias) and Model (2) random allocations.

	Dependent variable:			
	Bias: p(favored ∣ nonrandom)		Impartiality: $p(random)$	
	(1)		(2)	
Constant	1.187$^+$	(0.657)	−0.087	(0.366)
p(non-random∣individual)	−0.125***	(0.018)		
p(non-random∣feature)	0.138	(0.107)		
Feature: Flu vaccination	−0.155	(0.217)	0.166	(0.363)
Flu vaccination behavior	−0.075	(0.069)	−0.164*	(0.068)
Last 2 timepoints	−0.243***	(0.064)	−0.556***	(0.062)
Attitude: Flu vaccination (z)	0.267***	(0.070)	−0.074	(0.070)
Feature: Flu vaccination × Flu vaccination behavior	0.082	(0.054)	0.006	(0.040)
Feature: Flu vaccination × Last 2 timepoints	0.107*	(0.050)	−0.117**	(0.037)
Flu vaccination behavior × Last 2 timepoints	−0.058	(0.069)	0.120$^+$	(0.068)
Feature: Flu vaccination × Attitude: Flu vaccination (z)	0.154**	(0.056)	−0.091*	(0.042)
Last 2 timepoints × Attitude: Flu vaccination (z)	−0.088	(0.070)	−0.062	(0.070)
Feature: Flu vaccination × Flu vaccination behavior × Last 2 timepoints	−0.035	(0.054)	0.121**	(0.040)
Feature: Flu vaccination × Last 2 timepoints × Attitude: Flu vaccination (z)	−0.013	(0.056)	0.025	(0.042)
$\sigma_{participant}$	1.192		1.686	
$\sigma_{feature}$	0.406		0.706	
AIC	13357.09		28035.93	
Nakagawa's-R^2	0.07		0.03	
$LR\text{-}\chi^2$	96.92***		124.64***	
N	13065		25878	
n_{ind}	1298		1362	
Note:	$^+$p < 0.1; *p < 0.05;**p < 0.01; ***p < 0.001.			

0.001] with an interaction with later timepoints [$\chi^2(1) = 4.353$, $p = 0.037$)]. Organ donors tended to be more impartial across other features ($OR = 1.27$, $p = 0.044$, but not at the later timepoints $OR = 0.77$, $p = 0.142$), whereas they were consistently less impartial when patients differed in their organ donation status ($OR = 0.44$, $p < 0.001$ and $OR = 0.49$, $p = 0.014$). The difference in change from other features to organ donation was substantive in the first 4 timepoints ($OR_{difference} = 0.34$, $p < 0.001$) and pointed into the same direction at the later timepoints ($OR_{difference} = 0.64$, $p = 0.064$), despite overall lower impartiality. Thus, it appears that participants who were willing to donate their organs favored patients who were described to be similar to them in this regard.

Vaccination Next, we considered the effects of patients' and participants attitudes and behaviors towards flu vaccination. A model including feature-specific interactions for flu vaccination attitude and self-reported vaccination behavior reveal feature specific effects on bias for participants attitude towards flu vaccination [$\chi^2(1) = 7.667$, $p = 0.006$, robust to timepoint of

Supplementary Table 9. Estimates from a logistic mixed model predicting allocations to the female patient as a function of participants' gender. Model (1) predicts allocations to the the female patient among non-random allocations (bias) and Model (2) random allocations.

	Dependent variable:			
	Bias: p(favored ∣ nonrandom)		Impartiality: $p(random)$	
	(1)		(2)	
Constant	1.385*	(0.657)	0.350	(0.345)
p(non-random∣individual)	−0.123***	(0.018)		
p(non-random∣feature)	0.141	(0.116)		
Feature: Sex	0.033	(0.238)	0.578^{+}	(0.341)
Gender: Female	0.357***	(0.075)	0.220***	(0.064)
Last 2 timepoints	−0.241**	(0.076)	−0.630***	(0.064)
Feature: Sex × Gender: Female	0.211***	(0.063)	0.006	(0.039)
Feature: Sex × Last 2 timepoints	0.126*	(0.063)	−0.184***	(0.039)
Gender: Female × Last 2 timepoints	0.044	(0.075)	−0.027	(0.064)
Feature: Sex × Gender: Female × Last 2 timepoints	0.053	(0.063)	0.016	(0.039)
$\sigma_{participant}$	1.206		1.678	
$\sigma_{feature}$	0.41		0.656	
AIC	13358.86		28026.52	
Nakagawa's-R^2	0.06		0.05	
LR-χ^2	87.15***		126.05***	
N	13065		25878	
n_{ind}	1298		1362	
Note:	$^{+}$p < 0.1; *p < 0.05;**p < 0.01; ***p < 0.001.			

data collection $\chi^2(1) = 0.056$, $p = 0.813$], without additional effects of their behavior [$\chi^2(1) = 2.270$, $p = 0.132$, robust to timepoint of data collection $\chi^2(1) = 0.403$, $p = 0.526$]. Relative to other patient features, bias towards the patient vaccinated against the flu increased more strongly with a more positive attitude towards flu vaccination ($\beta_{diff} = 0.31$, $p = 0.006$). Concerning impartiality, we find evidence for a specific effect of participants attitude [$\chi^2(1) = 4.747$, $p = 0.029$, robust to timepoint of data collection $\chi^2(1) = 0.353$, $p = 0.553$] without overall evidence for a specific effect of behavior [$\chi^2(1) = 0.025$, $p = 0.875$], but a moderating influence of timepoint of data collection [$\chi^2(1) = 9.163$, $p = 0.002$]. In line with a role of attitude, impartiality decreased with a more positive attitude towards flu vaccination ($\beta_{diff} = -0.18$, $p = 0.029$). The results for self-reported vaccination behavior on impartiality were less clear:

Whereas participants who reported being vaccinated against the flu tended were less impartial across vaccination-unrelated patient features both in the first 4 ($OR = 0.71$, $p = 0.016$) and marginally in the last 2 timepoints ($OR = 0.71$, $p = 0.061$), they were even less impartial when patients differed in whether they were vaccinated against the flu in the first 4 timepoints ($OR = 0.45$, $p = 0.001$) but clearly not in the last 2 timepoints ($OR = 1.18$, $p = 0.575$). This corresponds to a stronger decrease in impartiality relative to other features in the first 4 timepoints ($OR_{diff} = 0.63$, $p = 0.021$) but even an *increase* in impartiality in the latter 2 ($OR_{diff} = 1.67$, $p = 0.042$). Thus, whereas we find clear evidence for ingroup favoritism among the vaccinated in the first 4 timepoints, we do not find this in the latter timepoints where vaccinated individuals appear to be even more impartial towards patients who are unwilling to get vaccinated against the flu compared to other features. Overall, this is evidence that an individual's attitude towards flu vaccination is a strong predictor of whether a vaccinated patient is more likely to be saved, replicating and extending previous work by[14]. Conversely, it is less clear whether vaccination behavior constitutes a marker of group membership that is relevant for discrimination beyond attitude.

Patient sex In line with well-established biases, we also find in-group favoritism based on *demographics and group membership*. We find indication of sexism. Bias towards the female patient specifically varied with participant sex [$\chi^2(1) = 11.383$, $p = 0.001$], whereas there was no evidence for differences in impartiality impartiality did not [$\chi^2(1) = 0.021$, $p = 0.884$]. Although females were also more biased than males towards the favored patient across other features [$OR = 1.34$, $p = 0.002$], this difference in bias was even more pronounced when patients differed by sex [$OR = 3.12$, $p < 0.001$, $OR_{difference} = 2.33$, $p = 0.001$]. Absent evidence for moderating influence of timepoint of data collection on bias [$\chi^2(1) = 0.727$, $p = 0.394$] or impartiality [$\chi^2(1) = 0.174$, $p = 0.677$] indicates robustness of these findings. This indicates that overall favoritism for female patients is increased among females and largely removed among males.

Ethnicity When deciding based on patient ethnicity, we find evidence for feature-specific differences in bias [$\chi^2(2) = 13.286$, $p = 0.001$] and impartiality [$\chi^2(2) = 11.749$, $p = 0.003$] as a function of participant ethnicity. Participants reporting to be African American were less biased towards the favored patient across features than white participants ($OR = 0.47$, $p < 0.001$) or other ethnicities ($OR = 0.50$, $p < 0.001$), with no difference between white participants and other ethnicities ($OR = 0.94$, $p = 0.973$). However, when patients differed in their ethnicity, the decrease in bias among African Americans was not more pronounced compared to whites ($OR = 0.73$, $p = 0.903$) but compared to other ethnicities ($OR = 0.23$, $p < 0.001$), also with

Supplementary Table 10. Estimates from a logistic mixed model predicting allocations to the female patient as a function of participants' gender. Model (1) predicts allocations to the the female patient among non-random allocations (bias) and Model (2) random allocations.

	Dependent variable:			
	Bias: p(favored | nonrandom) (1)		Impartiality: $p(random)$ (2)	
Constant	0.931	(0.663)	0.306	(0.334)
p(non-random|individual)	-0.112^{***}	(0.018)		
p(non-random|feature)	0.142	(0.118)		
Feature: Ethncity	-0.209	(0.250)	0.692^{*}	(0.327)
African American	-0.534^{***}	(0.129)	-0.558^{***}	(0.120)
Other ethnicity	-0.004	(0.176)	0.322^{*}	(0.151)
Last 2 timepoints	-0.182^{+}	(0.100)	-0.529^{***}	(0.088)
Feature: Ethncity × African American	-0.053	(0.108)	-0.184^{*}	(0.072)
Feature: Ethncity × Other ethnicity	-0.275^{+}	(0.152)	0.147	(0.093)
Feature: Ethncity × Last 2 timepoints	0.156^{+}	(0.085)	-0.208^{***}	(0.054)
African American × Last 2 timepoints	0.138	(0.129)	0.177	(0.120)
Other ethnicity × Last 2 timepoints	-0.013	(0.176)	-0.048	(0.151)
Feature: Ethncity × African American × Last 2 timepoints	-0.037	(0.108)	0.114	(0.072)
Feature: Ethncity × Other ethnicity × Last 2 timepoints	0.140	(0.152)	-0.143	(0.093)
$\sigma_{participant}$	1.167		1.675	
$\sigma_{feature}$	0.411		0.631	
AIC	13327.73		28019.41	
Nakagawa's-R^2	0.08		0.06	
$LR\text{-}\chi^2$	126.29^{***}		141.16^{***}	
N	13065		25878	
n_{ind}	1298		1362	
Note:	$^{+}$p < 0.1; *p < 0.05;**p < 0.01; ***p < 0.001.			

higher bias among whites than among other ethnicities ($OR = 3.14$, $p = 0.045$). The change relative to non-ethnicity features differed between African American participants and whites ($OR = 0.47$, $p = 0.015$), whites and other ethnicities ($OR = 0.30$, $p = 0.026$), but not between African Americans and other ethnicities ($OR = 0.64$, $p = 0.745$). Although Figure 2 shows reversed bias towards the white patient among non-whites, the bias is estimated to be decreased but not reversed after controlling for general response tendencies [$bias(white) = 0.56$, $p = 0.622$].

Moreover, participants reporting to be African American were less impartial towards the favored patient across features than white participants ($OR = 0.58$, $p = 0.030$) or other ethnicities ($OR = 0.56$, $p < 0.001$), with no difference between white participants and participants from other ethnicities ($OR = 1.03$, $p = 0.999$). However, when patients differed in their ethnicity, the decrease in impartiality among African Americans tended to be more pronounced compared to whites ($OR = 0.30$, $p = 0.003$) and other ethnicities ($OR = 0.36$, $p < 0.001$). An effect suggesting lower impartiality among whites than among other ethnicities cannot be distinguished from chance in the current data ($OR = 0.82$, $p = 0.906$), likely because of few observations of the "other category". The change in the effect of participant ethnicity from non-ethnicity fatures to other features differed marginally between African American participants and Whites ($OR = 0.64$, $p = 0.061$) and between African Americans and other ethnicities ($OR = 1.94$, $p = 0.097$) but not between Whites and other ethnicities ($OR = 1.25$, $p = 0.812$).

Absent evidence for moderating influence of timepoint of data collection on bias [$\chi^2(2) = 1.220$, $p = 0.543$] or impartiality [$\chi^2(2) = 2.762$, $p = 0.251$] indicates robustness of these findings. Thus, white participants were more biased than participants reporting to be African American, both overall but specifically when patients differed in their ethnicity. Thereby, African American participants tended to be less impartial, with suggestive evidence that this was stronger when patients differed in their ethnicity.

Religiosity Despite overall high impartiality, decisions between patients based on their belief in god exhibit clear in-group favoritism. We find feature-specific interactions for bias ($\chi^2(1) = 74.060$, $p < 0.001$) and impartiality ($\chi^2(1) = 49.554$, $p < 0.001$). There is a moderating influence of the timepoint of data collection on bias ($\chi^2(1) = 3.076$, $p = 0.079$) and impartiality ($\chi^2(1) = 4.225$, $p = 0.040$). As also Supplementary Table 11, shows, with participants' self-reported belief in god bias increased when patients differed in their belief in god relative to other features ($\beta_{diff} = -1.67$, $p < 0.001$, although slightly weakened at later timepoints, $\beta_{diff} = -1.10$, $p < 0.001$) whereas impartiality decreased with belief in god in general (first 4 timepoints: $\beta_{diff} = 0.48$, $p < 0.001$), even more when patients differed in their belief in god and in the later timepoints ($\beta_{diff} = 0.88$, $p < 0.001$).

Political orientation (and foreigners) Discrimination of patients with migration background varies as a function of participants' political orientation. Beyond immediate group membership, political orientation affected how participants decided between U.S.-born patients and migrants. When patients with a migration background had to be traded off with a a U.S.-born

Supplementary Table 11. Estimates from a logistic mixed model predicting allocations to the patient who believed in god as a function of participants' religiosity. Model (1) predicts allocations to the the patient who believed in god among non-random allocations (bias) and Model (2) random allocations.

	Dependent variable:			
	Bias: p(favored ∣ nonrandom)		Impartiality: $p(random)$	
	(1)		(2)	
Constant	0.863	(0.641)	0.443	(0.353)
p(non-random∣individual)	-0.061^{***}	(0.018)		
p(non-random∣feature)	0.081	(0.110)		
Feature: Belief in god	-0.640^{**}	(0.230)	0.597^{+}	(0.350)
Religiosity (z-standardized)	0.012	(0.092)	-0.903^{***}	(0.072)
Last 2 timepoints	0.024	(0.089)	-0.273^{***}	(0.069)
Feature: Belief in god × Religiosity (z-standardized)	0.692^{***}	(0.080)	-0.342^{***}	(0.049)
Feature: Belief in god × Last 2 timepoints	0.220^{**}	(0.078)	−0.017	(0.046)
Religiosity (z-standardized) × Last 2 timepoints	-0.202^{*}	(0.091)	-0.212^{**}	(0.072)
Feature: Belief in god × Religiosity (z-standardized) × Last 2 timepoints	-0.141^{+}	(0.080)	-0.100^{*}	(0.049)
$\sigma_{participant}$	1.093		1.612	
$\sigma_{feature}$	0.398		0.673	
AIC	13077.41		27884.44	
Nakagawa's-R^2	0.13		0.08	
$LR\text{-}\chi^2$	368.61^{***}		268.13^{***}	
N	13065		25878	
n_{ind}	1298		1362	
Note:	$^{+}$p < 0.1; *p < 0.05;**p < 0.01; ***p < 0.001.			

Supplementary Table 12. Estimates from a logistic mixed model predicting allocations to the patients who recently arrived with a work permit and recently sought refuge as a function of participants' political orientation. Model (1) predicts allocations to the the patients who recently arrived with a work permit and the patient who recently sought refuge among non-random allocations (bias) and Model (2) random allocations.

	Dependent variable:			
	Bias: p(favored ∣ nonrandom)		Impartiality: $p(random)$	
	(1)		(2)	
Constant	1.628^{*}	(0.704)	−0.304	(0.505)
p(non-random∣individual)	-0.133^{***}	(0.018)		
p(non-random∣feature)	0.144	(0.104)		
Feature: Migration with permit	0.131	(0.211)	−0.043	(0.367)
Conservatism (z-standardized)	0.112	(0.078)	-0.603^{***}	(0.071)
Last 2 timepoints	-0.403^{***}	(0.083)	-0.320^{***}	(0.071)
Feature: Took refuge	0.187	(0.211)	−0.065	(0.367)
Feature: Migration with permit × Conservatism (z-standardized)	0.010	(0.049)	-0.210^{***}	(0.037)
Feature: Migration with permit × Last 2 timepoints	−0.012	(0.052)	0.048	(0.037)
Conservatism (z-standardized) × Last 2 timepoints	−0.032	(0.078)	0.201^{**}	(0.071)
Conservatism (z-standardized) × Feature: Took refuge	0.037	(0.050)	-0.149^{***}	(0.037)
Last 2 timepoints × Feature: Took refuge	−0.036	(0.053)	0.087^{*}	(0.036)
Feature: Migration with permit × Conservatism (z-standardized) × Last 2 timepoints	0.010	(0.049)	0.141^{***}	(0.037)
Conservatism (z-standardized) × Last 2 timepoints × Feature: Took refuge	−0.059	(0.050)	0.157^{***}	(0.037)
$\sigma_{participant}$	1.207		1.681	
$\sigma_{feature}$	0.397		0.713	
AIC	13388.83		27942.15	
Nakagawa's-R^2	0.06		0.04	
$LR\text{-}\chi^2$	65.19^{***}		218.42^{***}	
N	13065		25878	
n_{ind}	1298		1362	
Note:	$^{+}$p < 0.1; *p < 0.05;**p < 0.01; ***p < 0.001.			

patient, more conservative political orientation was not specifically related to bias towards migrants with a work permit [$\chi^2(1) = 0.063$, $p = 0.802$] or refugees [$\chi^2(1) = 0.626$, $p = 0.429$, and largely unrelated to bias overall, see Supplementary Table 12]. However, political orientation was specifically related to impartiality towards migrants with a work permit [$\chi^2(1) = 49.205$, $p < 0.001$] and refugees [$\chi^2(1) = 29.829$, $p < 0.001$)].

Although compared to the remaining features we find a more negative trend on impartiality for both the refugee ($\beta_{diff} = -0.61$, $p < 0.001$) and the work permit holder ($\beta_{diff} = -0.70$, $p < 0.001$) throughout the first 4 timepoints of data collection, in the last 2 timepoints the effect for the refugee disappears ($\beta_{diff} = 0.01$, $p = 0.900$) and the effect for the work permit holder diminshes so far that we at least cannot detect it anymore ($\beta_{diff} = -0.14$, $p = 0.249$).

This extends the finding by[15] that patient nationality was more relevant among more conservative decision makers. Additionally, our results suggest that political orientation and own migration status may act on different facets of the decision,

namely, how many show a bias and how strong the bias is. However, the result is only robust to the first 4 timepoints of data collection, which could indicate a change in the perceptions, less careful responding and should be scruitinzed by future work.

References

1. Awad, E. *et al.* The moral machine experiment. *Nature* **563**, 59–64, DOI: 10.1038/s41586-018-0637-6 (2018).
2. Barbor, T. F., Higgins-Biddle, J. C., Saunders, J. B. & Montero, M. G. *AUDIT-the alcohol use disorders identification test. Guidelines for Use in Primary Care.* (WHO, 2001).
3. Falk, A., Becker, A., Dohmen, T. J., Huffman, D. & Sunde, U. The preference survey module: A validated instrument for measuring risk, time, and social preferences (2016).
4. Falk, A. & Hermle, J. Relationship of gender differences in preferences to economic development and gender equality. *Science* **362**, DOI: 10.1126/science.aas9899 (2018). https://science.sciencemag.org/content/362/6412/eaas9899.full.pdf.
5. Betsch, C. *et al.* The four weeks before lockdown during the COVID-19 pandemic in germany: a weekly serial cross-sectional survey on risk perceptions, knowledge, public trust and behaviour, 3 to 25 March 2020. *Eurosurveillance* **26**, DOI: https://doi.org/10.2807/1560-7917.ES.2021.26.42.2001900 (2021).
6. Arechar, A. A. & Rand, D. G. Turking in the time of COVID. *Behav. Res. Methods* **53**, 2591–2595, DOI: 10.3758/s13428-021-01588-4 (2021).
7. R Core Team. *R: A Language and Environment for Statistical Computing*. R Foundation for Statistical Computing, Vienna, Austria (2021).
8. Bates, D., Maechler, M., Bolker, B. & Walker, S. *lme4: Linear Mixed-Effects Models using Eigen and S4* (2021). R package version 1.1-27.1.
9. Fox, J. & Weisberg, S. *An R Companion to Applied Regression* (Sage, Thousand Oaks CA, 2019), third edn.
10. Agresti, A. *Categorical data analysis* (Wiley-Interscience, Hoboken, NJ, 2002), 2. edn.
11. Jaeger, T. F. Categorical data analysis: Away from ANOVAs (transformation or not) and towards logit mixed models. *J. Mem. Lang.* **59**, 434–446, DOI: 10.1016/j.jml.2007.11.007 (2008).
12. Wald, A. Tests of statistical hypotheses concerning several parameters when the number of observations is large. *Transactions Am. Math. Soc.* **54**, 426–482 (1943).
13. Lenth, R. V. *emmeans: Estimated Marginal Means, aka Least-Squares Means* (2022). R package version 1.7.2.
14. Sprengholz, P., Korn, L., Felgendreff, L., Eitze, S. & Betsch, C. A lay perspective on prioritization for intensive care in pandemic times: Vaccination status matters. *Clin. Ethics* **0**, 14777509221094474, DOI: 10.1177/14777509221094474 (2022).
15. Jin, L., Huang, Y., Liang, Y. & Zhang, Q. Who gets the ventilator? Moral decision making regarding medical resource allocation in a pandemic. *J. Assoc. for Consumer Res.* **6**, 159–167, DOI: 10.1086/711734 (2021).
